# Chemical Insights into Interfacial Materials from Brazilian Crude Oils via Comparative Extraction Methods

**DOI:** 10.1021/acs.langmuir.6c00674

**Published:** 2026-06-01

**Authors:** Thaynara R. S. Costa, Bruna F. Cavalini, Luciara Costa de Souza, Marcos Henrique Oliveira Petroni, Lays Rafalscky, Amanda Eiriz Feu, Boniek Gontijo, Rogério Mesquita de Carvalho, Osvaldo Karnitz, Luiz Carlos do C. Marques, Marcia Cristina Khalil de Oliveira, Eliane Valéria de Barros, Renato do Nascimento Siqueira, Vitor C. B. Pegoretti, Lindamara Maria de Souza, Wanderson Romão

**Affiliations:** † 28126 Federal University of Espírito Santo, Vitória 29075-910, Brazil; ‡ Federal Institute of Education, Science and Technology of Espírito Santo, Vitória 29040-780, Brazil; § 67824 Federal University of Goias, Goiania 74.690-631, Brazil; ∥ 42506 Petróleo Brasileiro S.A., CENPES, Rio de Janeiro 21941-915, Brazil; ⊥ Federal Institute of Education, Science and Technology of Espírito Santo, São Mateus 29932-540, Brazil; # Federal Institute of Education, Science and Technology of Espírito Santo, Vila Velha 29075-910, Brazil

## Abstract

The formation and stability of water-in-oil (W/O) emulsions in petroleum are strongly influenced by species that accumulate at the oil–water interface, collectively referred to as interfacial material (IM). Understanding the composition of IM is essential for clarifying the mechanisms responsible for emulsion stability and interfacial rigidity, although its high chemical complexity makes the identification of its major constituents challenging. In this study, IM fractions from four Brazilian crude oils were isolated using two methods, centrifugation followed by Dean–Stark distillation (IMR) and adsorption on wet silica (IMW), and then characterized by negative-ion electrospray Fourier-transform ion cyclotron resonance mass spectrometry (ESI(−) FT-ICR MS), gas chromatography with flame ionization detection (GC–FID), elemental analysis (CHNS-O), and mid-Fourier transform infrared spectroscopy (mid-FTIR). Their ability to stabilize emulsions was also evaluated. The results showed that both extraction methods enriched the IM fractions with highly polar oxygenated classes and reduced the relative contribution of pyrrolic species (N­[H]) compared with the original crude oils. The wet silica method was more selective for highly polar compounds, particularly the O_2_[H] class, commonly associated with naphthenic acids and rigid interfacial films. In contrast, the centrifugation/Dean–Stark method recovered an interfacial fraction with broader compositional coverage, including lower-polarity classes such as NO_2_[H] and NO_3_[H], where GC–FID, elemental analysis, and FTIR analyses also confirmed a more aliphatic character for the IMR fractions obtained by centrifugation. Model emulsion tests further showed that the IM fractions obtained by centrifugation/Dean–Stark conferred greater stability to the W/O system than those isolated using wet silica, indicating that emulsion stabilization depends on the combined action of polar and nonpolar species.

## Graphical abstract

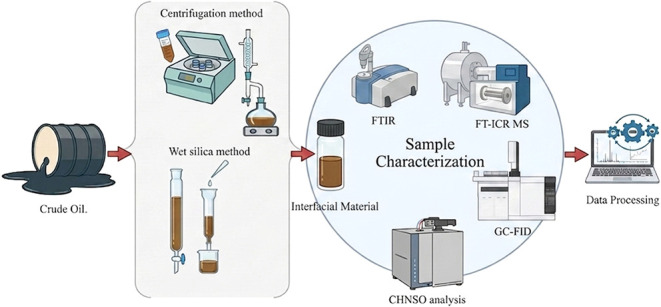

## Introduction

During extraction, crude oil and produced water are subjected to intense shear in production flowlines, which promotes the formation of emulsions. An emulsion is a colloidal dispersion composed of two immiscible phases, in which one phase is dispersed within the other.^1,2^ Stable emulsions are undesirable because they can substantially increase operational and production costs,^3^ in addition to altering the rheological properties of the fluid, as well as promote corrosive processes, hydrate formation, and scale deposition in production facilities^4,5^

The formation and stability of crude oil emulsions are governed by multifactorial mechanisms. Among them are intermolecular and interfacial forces between droplets, together with the adsorption of asphaltenes, resins, naphthenic acids (NAs),^1^ and fine solids at the water–oil (W/O) interface, that lead to the formation of an interfacial film. This film coats the droplets and increases the emulsion’s kinetic stability.^3^ In other words, although emulsions are thermodynamically unstable, their formation and persistence in crude oil are directly influenced by the composition of the interfacial material (IM), which consists of species that accumulate at the interface.^4^ Consequently, the molecular composition of the IM determines emulsion stability and helps identify the chemical species that contribute most significantly to the formation of the interfacial layer.^5^ Thus, elucidating the chemical composition of the IM in crude oil is crucial for understanding the mechanisms responsible for emulsion stability and interfacial rigidity.^6^

The complex nature of crude oil makes it difficult to determine which compounds or classes of compounds are responsible for emulsion behavior. Therefore, a common approach has been to fractionate the crude oil (i.e., to isolate compound classes) by selective extraction or chromatographic separation and subsequently to analyze the isolated fractions using analytical techniques such as Fourier transform ion cyclotron resonance mass spectrometry (FT-ICR MS).^1,4^

Other studies have proposed complementary approaches to elucidate the chemical composition and role of the IM, including size-exclusion chromatography coupled with high-resolution inductively coupled plasma mass spectrometry (GPC-ICP-HR-MS) for the speciation of metals associated with heteroatoms,^7^ and ion mobility mass spectrometry with collision-induced dissociation (IM-MS/CID) to resolve functional isomers.^1^ In addition, small-angle X-ray scattering (SAXS) and time-domain nuclear magnetic resonance (TD-NMR) have been employed to investigate the colloidal organization and physicochemical characteristics of the IM in emulsion systems,^9^ whereas Fourier transform infrared spectroscopy in attenuated total reflection mode (FTIR-ATR) has been used to identify functional groups and spectral coefficients.^8,10^ The use of different analytical techniques has enabled significant advances in understanding how complex polar fractions, as well as interfacial mineral solids adsorbed at the interface can contribute to emulsions formation and stabilization.

Isolation of the IM from crude oil and its subsequent characterization makes it possible to correlate its chemical composition with emulsion stability.^7^ The IM of W/O emulsions can be isolated by artificially emulsifying water in oil, followed by the collection of the water droplets, carrying adsorbed IM, by sedimentation or centrifugation. However, despite the relatively high yield, this procedure still leads to contamination with other constituents.^6^ To overcome this limitation, Wu (2003) developed the “heavy water method”. In this procedure, the emulsion is dispersed in Heptol and centrifuged, with the unique feature of using deuterium oxide (D_2_O) instead of ordinary water (H_2_O). This strategy reduces the likelihood of IM contamination, but it is unable to separate interfacial mineral solids, which sediment in the same manner as the D_2_O droplets. This is disadvantageous, since studies in the literature have shown that mineral solids are among the components that can stabilize W/O emulsions.^11,12^

To increase the purity of the isolated IM, Wu (2008) proposed the “selective cream” method, which involves modifying the density of the hydrocarbon phase and adding a layer of clean D_2_O on top. In this procedure, during centrifugation, H_2_O droplets containing IM are driven upward through the D_2_O layer, while noninterfacially active organics are retained below the D_2_O layer and noninterfacial solids sink into the oil phase. In this way, contaminants are “selectively” removed from the isolated IMs. However, the results suggested that the interfacial organic material constitutes the main emulsion stabilizer, whereas no single organic species dominates the interface.^12^

In contrast, the “wet silica” adsorption method proposed by Jarvis et al. (2015) has been introduced as a simpler and more cost-effective IM separation approach and has been widely employed in the literature.^5,7–9,13,14^ This method relies on the hydrophilicity of the target species, which are strongly adsorbed onto the surface water layer, and IM separation is achieved by elution with different polar solvents.^1,14^ Nevertheless, it is restricted to crude oil samples and yields only small amounts of IM, which may limit analytical possibilities and the exploration of correlations in exploratory studies. According to results reported in the literature, IM isolated either by the heavy-water method or by wet silica and characterized by FT-ICR MS shows predominant enrichment in oxygenated and sulfur-containing acidic classes, particularly O_2_, O_4_, O_3_S, and O_4_S, suggesting that these functional groups are primarily responsible for interfacial activity.

Another approach for IM isolation was presented by Pereira et al. (2014), who employed centrifugation followed by Dean–Stark distillation.^15^ In this method, preformed emulsions are subjected to centrifugation to promote separation of the aqueous, oil, and unresolved phases. The unresolved phase is combined with a volume of xylene and subjected to Dean–Stark distillation. Subsequently, xylene is removed using a rotary evaporator (Rotavapor) to recover the solvent and obtain the IM. This procedure also enables the recovery of insoluble salts present in the emulsion; however, it involves a relatively lengthy analytical workflow. Using ESI(−)­FT-ICR MS, the authors identified a predominance of the N class in the emulsified phase, in addition to phenolic compounds (O class) and acidic species (O_2_, O_4_, O_4_S, and NO/NO_2_ classes), highlighting the interfacial activity of molecules containing at least one oxygen atom,^10,15,16^

In 2025, Souza et al. showed, by ESI(−) FT-ICR MS, that the IM isolated from a naturally emulsified crude oil using a centrifugation-based methodology was enriched in oxygenated classes, particularly naphthenic acids and mixed heteroatomic species. The N_x_O_y_S_u_[H] notation, where x, y, and u indicate the number of nitrogen, oxygen, and sulfur atoms, respectively, assigned to the molecular formula, while [H] symbol represents deprotonated species detected in negative ionization mode. Based on this classification, the authors observed that highly aromatic compounds migrated to the NO_2_[H] and NO_3_[H] classes, whereas both linear and aromatic naphthenic acids (O_2_[H] class) became more prominent.^16^ Similarly, Petroni et al. (2025) used mid-Fourier transform infrared (mid-FTIR) spectroscopy and elemental (CHNS-O) analysis to characterize the IM, that presented higher aromaticity (∼1606 cm^–1^), enhanced carbonyl (∼1700 cm^–1^) and sulfoxide bands, as well as higher CH_3_/CH_2_ ratios relative to the parent crude oil.^10^

Although previous studies have characterized IM isolated from crude oils, the extent to which the isolation methodology influences the interpretation of its chemical composition and its relationship with emulsion stability remains not fully understood.^17^ In particular, the literature still lacks a detailed discussion of how different methods may selectively recover distinct subsets of the interfacial material.

In this context, the novelty of this study lies in the direct and multitechnique comparison between wet silica adsorption and centrifugation/Dean–Stark extraction, applied to the same set of four Brazilian crude oils. The isolated fractions were characterized by ESI(−)­FT-ICR MS, chromatography with flame ionization detection (GC-FID), CHNS-O analysis, and mid-FTIR spectroscopy, and were also evaluated for their ability to stabilize model W/O emulsions. The results demonstrate that the methods isolate chemically distinct fractions, with differences in molecular composition, aromaticity, and in the distribution of O_2_[H] species. Overall, this study highlights that methodological selectivity impacts the interpretation of interfacial composition and the mechanisms involved in emulsion stabilization.

## Materials and Methods

### Chemicals

All chemicals used for the development of the extraction methods for IM isolation were of analytical grade (purity >99.5%), and those employed in the FT-ICR MS and GC-FID analyses were of HPLC grade. Xylene and heptane were obtained from Neon Comercial Reagentes Analíticos Ltda (Suzano, SP, Brazil). Toluene and methanol were supplied by Sigma-Aldrich Brasil Ltda (Duque de Caxias, RJ, Brazil). Sodium trifluoroacetate (98%, NaTFA) and ammonium hydroxide (28%, NH_4_OH) were purchased from Sigma-Aldrich Chemicals (St. Louis, MO, USA). All reagents used in the analyses were employed as received, without any further purification.

### Oil Characterization

In this study, four crude oil samples from producing fields located in sedimentary basins along the Brazilian coast, labeled “B”, “C”, “D”, and “F”, were provided by CENPES/PETROBRAS (Rio de Janeiro, Brazil).

The oils were characterized according to the methodology described by Souza et al. (2025) and Petroni et al. (2025).^10,16^ The physicochemical properties of the dehydrated oils were evaluated (Table S1) in accordance with the relevant American Society for Testing and Materials (ASTM) standard methods, including basic sediment and water (BS&W, ASTM D4007),^18^ water content in oil after dehydration (ASTM D4377),^19^ density at 20 °C (ASTM D5002),^20^ American Petroleum Institute (API) gravity (ASTM D1250),^21^ dynamic viscosity at 60 °C (ASTM D7042),^22^ total acid number (TAN, ASTM D664),^23^ total salinity index (TSI) (ASTM D6470),^23^ pH of the dehydration and wash waters (pH measurement), interfacial tension by the pendant drop method,^24^ average droplet-size distribution^24^ and contents of saturates, aromatics, and polars (SAP, modified ASTM D2549).^25^

### IM Isolation

The B, C, D, and F crude oil samples were subjected to IM extraction by two different methods: (i) wet silica adsorption, as proposed by Jarvis et al. (2015) and adapted by Norrman et al. (2020), and (ii) centrifugation/Dean–Stark, according to the methodology of Pereira et al. (2014). To assess the chemical species present in the IM obtained by each method, the crude oils and their corresponding IM fractions were characterized by ESI(−)­FT-ICR MS, GC-FID, CHNS-O, and mid-FTIR,^4,5,15^

For the wet silica extraction, approximately 15 g of dehydrated crude oil were mixed with 15 g of water-saturated silica (pH ∼ 7). Then, 250 mL of Heptol (*n*-heptane/toluene, 50:50 v/v) were added, and the mixture was subjected to open-column chromatography with two sequential elution steps: a cleanup (CU) step with Heptol to remove weakly adsorbed compounds, followed by elution with toluene/methanol (70:30 v/v) to recover the interfacially active material (IM). The remaining solvent was evaporated at room temperature to obtain the wet silica interfacial material (IMW).^4^

For IM extraction by the centrifugation/Dean–Stark method, approximately 100 g of emulsified crude oil (with its own formation water) were processed in a Rotina 420 R/H centrifuge (Hettich, Tuttlingen, Germany) at 9500 rpm (RCF = 12,007 × g) for 120 min at 60 °C; these operating conditions were optimized in previous work.^10,16^ This process separates the oil phase from the unresolved emulsion phase (emulsion phase of residue, EPR). Subsequently, about 2 g of the EPR fraction were transferred to a round-bottom flask and mixed with 80 mL of xylene. The mixture was subjected to Dean–Stark distillation, during which the xylene-water azeotrope was separated. The water-free extract was then centrifuged to remove any insoluble salts. Finally, xylene was removed by rotary evaporation, yielding the interfacial material of the residue (IMR).^15^ Figure 1 presents a general schematic of IM isolation in this study.

**Figure 1:**
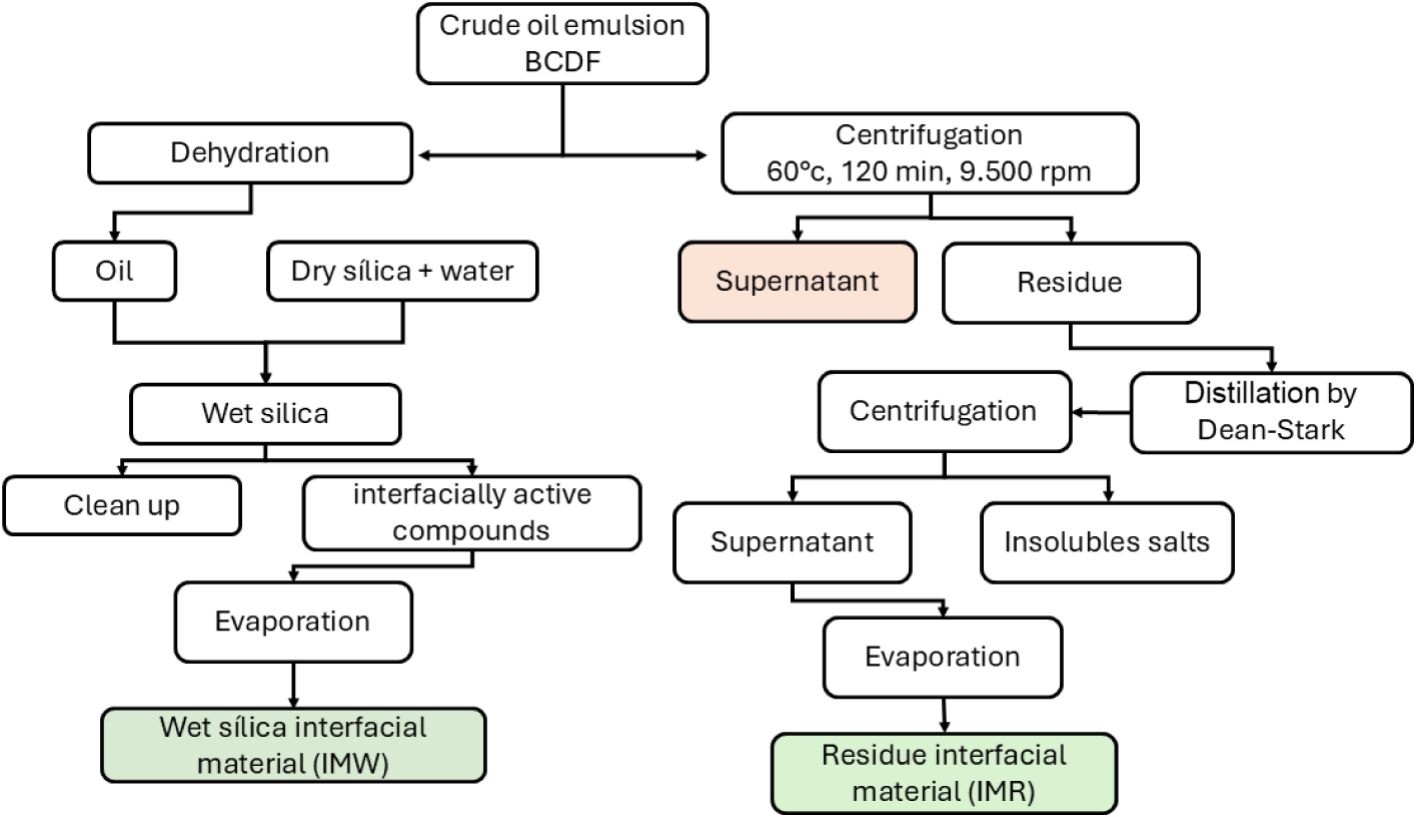
Isolation of IM and other fractions from crude oils B, C, D, and F using the wet silica and centrifugation/Dean–Stark methodologies.

### IM Characterization

Analyses were performed on a 7 T Solarix FT-ICR mass spectrometer (Bruker Daltonics, Bremen, Germany) operated over a dynamic ion acquisition range of *m*/*z* 100–2000. The resolving power (m/Δm_50_%) was 850,000–880,000 at *m*/*z* 400, where Δm_50_% corresponds to the full peak width at half-maximum, and the mass accuracy was better than 1 ppm.

Samples were prepared at a concentration of 0.500 mg mL^–1^ in a toluene:methanol mixture (1:1, v/v) containing 50 μL of NH_4_OH and were directly infused into the electrospray ionization source operated in negative-ion mode (ESI(−)).

The instrument was externally calibrated prior to analysis using a NaTFA solution (*m*/*z* 100–2000), and the acquired spectra were subsequently recalibrated with DataAnalysis 4.0 (Bruker Daltonics) using the most abundant homologous series of alkylated compounds in each sample.^26,27^

Mass spectra were processed using Composer *software*,^28^ employing an assignment algorithm specifically developed for petroleum samples, from which molecular formulas, average molecular weights (Mw), and compound class distributions were obtained. The data were then exported and processed in Thanus *software*, developed at LaCEM-UFG,^29^ for compilation and visualization. Based on the assigned formulas, classical Petroleomic plots were constructed, including heteroatomic class distributions, double bond equivalent (DBE) versus relative abundance (intensity), DBE versus carbon number (CN), and Van Krevelen diagrams. The degree of unsaturation of each compound (DBE) was calculated using eq 1, with higher values indicating a greater hydrogen deficiency in the molecule.

1 $$DBE=C-\frac{H}{2}+\frac{N}{2}+1$$

where C, H, and N represent, respectively, the number of carbon (CN), hydrogen, and nitrogen atoms in the molecular formula of the analyzed compound.

IM samples were analyzed by GC-FID following the methodology described by Coutinho et al. (2018),^30^ with minor modifications. Approximately 10 mg of each sample were weighed directly into glass vials and dissolved in 1 mL of dichloromethane. Injections were performed in splitless mode (split ratio 1:20) using an HP-5MS fused-silica capillary column (30 m × 0.25 mm i.d., 0.25 μm film thickness, 5% phenyl–95% methylpolysiloxane) and helium as carrier gas (99.9999% purity). Analyses were carried out on a TRACE 1610 GC system coupled to an ISQ 7610 mass spectrometer (Thermo Fisher Scientific Inc., Waltham, MA, USA) equipped with a flame ionization detector (FID). The GC oven temperature was programmed from 40 to 320 °C at 6 °C min^–1^, then held at 310 °C for 10 min. The injector temperature was set at 310 °C and the detector temperature at 350 °C.

Elemental analysis was performed on an EA3100 analyzer (EuroVector, Pavia, Italy) based on the dynamic flash combustion technique to determine C, H, N, S, and O contents. Samples were encapsulated in tin (for CHNS) or silver (for O) capsules and combusted in dedicated reactors (980 °C for CHNS and 1080 °C for O) under a helium flow of 110 mL min^–1^ as carrier gas. The resulting gases were separated on dedicated chromatographic columns and detected by thermal conductivity detection (TCD). Elemental quantification was carried out by linear regression using calibration standards over a mass range of 0.5–1.5 mg.^10^

Mid-FTIR spectra (4000–400 cm^–1^) were acquired on an ALPHA II spectrometer (Bruker, Ettlingen, Germany) equipped with a QuickSnap ATR modular sampling system, using 16 scans per spectrum. Spectral data were processed in Origin *software* for peak organization and identification. Band-based spectral coefficients (C_n_) were determined from the maximum absorbance (optical density) values at wavenumbers close to the reference points described by Rakhmatullin et al. (2018), in order to summarize the main structural attributes.^10,31^ The spectral coefficient equations used to calculate from C_1_ to C_5_ are presented below.

2 $$C_{1}=\frac{D_{1600}}{D_{720}}$$

3 $$C_{2}=\frac{D_{1710}}{D_{1465}}$$

4 $$C_{3}=\frac{D_{1380}}{D_{1465}}$$

5 $$C_{4}=\frac{D_{720}+D_{1380}}{D_{1600}}$$

6 $$C_{5}=\frac{D_{1030}}{D_{1465}}$$

In this context, D_n_ denotes the optical density at wavenumber. Accordingly, the spectral coefficients were defined as follows: C_1_ (aromaticity) relates the absorbance of aromatic groups (D_1600_) to that of long methylene chains (D_720_, CH_2_ > 4); C_2_ (oxidation) compares the carbonyl absorbance (D_1710_) with methylene deformation (D_1465_); C_3_ (branching) accounts for methyl deformation (D_1380_) and the CH_3_/CH_2_ stretching ratio; C_4_ (aliphaticity) reflects the aliphatic content of long chains (e.g., CH_2_ near 720 cm^–1^); and C_5_ (sulfoxide) represents the contribution of S=O groups via D_1030_. These indices were used to compare IMR and IMW with the original crude oil.

### Emulsion Stability

To evaluate the efficiency of the interfacial materials (IM) in stabilizing emulsions, a model system was designed consisting of a commercial mineral oil as the oil phase and a saline solution (NaCl, 50 g L^–1^) as the aqueous phase, in a 50:50 v/v ratio. The interfacial materials used in these experiments were obtained from crude oil B. Three emulsions were prepared: (i) a control, without addition of IM; (ii) with IM obtained from crude oil B by the wet silica method (IMW); and (iii) with IM obtained from crude oil B by the centrifugation/Dean–Stark method (IMR), yielding three experimental emulsions. All emulsions were prepared following the same protocol. Initially, 125 g of the oil matrix were heated to 80 °C for 1 h. For the emulsions containing IM, 320 ± 50 mg of IM were then added under stirring for 24 h to ensure incorporation. In parallel, 125 g of the saline solution was heated to 60 °C for 1 h. The two phases (oil and aqueous) were then mixed and subjected to mechanical agitation (IKA T25 digital Ultra-Turrax) at 8000 rpm for 3 min, resulting in the final emulsions.

To assess the stability of the model emulsions containing IM, gravitational separation tests were first performed to evaluate W/O phase separation. Immediately after preparation (t = 0 h), approximately 50 mL of each emulsion were transferred to 50 mL conical polypropylene tubes and kept at room temperature (25 °C). Under these conditions, the emulsions were monitored at 0, 1, 24, 144, and 240 h. The percentage of separated water was calculated using eq 7 below.^32^

7 $$\%=\frac{Volumeofseparated\;water,\;mL}{Initial\;water\;volume,\;mL}\times 100$$

Complementary, accelerated sedimentation experiments were carried out using a LUMiSizer dispersion analyzer (LUM GmbH, Berlin, Germany) equipped with a near-infrared (NIR) light source (865 nm). The incident radiation enabled time-resolved monitoring of changes in the light transmission profile across the cuvette. For this purpose, 400 μL aliquots of each emulsion (t = 0 h) were individually transferred to polycarbonate cuvettes. The samples were subjected to centrifugation at 4000 rpm (Relative Centrifugal Force—RCF = 1.878 × g) for 66 min under two temperature conditions: 25 and 40 °C. Near-infrared transmission readings were recorded in 100 initial profiles at 5 s intervals, followed by 300 additional profiles at 10 s intervals.

## Results and Discussion

### Physicochemical Characterization

The characterization of the four crude oils is presented in Table S1, together with their physicochemical profiles. The crude oils analyzed exhibit BS&W contents in the range of 8–59% v/v, decreasing in the order B > D > C > F. Oil B showed a sediment content of 0.4% v/v. Owing to their BS&W levels, the crude oils display a typical W/O emulsion profile. Furthermore, according to the droplet size distribution (DSD), the emulsions formed by these crude oils tend to be highly stable.^33^

The salt content of the crude oils ranged from 3,500 to 16,646 mg kg^–1^, expressed as NaCl. Since salts and water are undesirable because they interfere with the determination of physicochemical properties, dehydration and desalting were required prior to physicochemical analyses and IM isolation by the wet silica method. After this step, the water content of the crude oils was reassessed to verify the efficiency of dehydration, yielding values lower than 0.5% v/v (ASTM D4377).^19^

As for the API gravity of the dehydrated oils, they can be classified as medium oils with values ranging from 25 to 28. Among the oils analyzed, crude oil D exhibited the highest API gravity (28.2), making it the lightest of the crude oils. All the crude oils showed a nonacidic nature, with a TAN lower than 0.5 mg KOH·g.^34^

Surface and interfacial tensions of the crude oils were also determined. The interfacial tension (mN m^–1^) was measured in two distinct media: a nonsaline medium consisting of deionized water and a saline medium consisting of formation water (salinity 1.80 × 10^–1^ mg L^–1^). The results showed that the interfacial tension values of the crude oils decreased in the saline medium, a behavior that indicates a greater tendency to form stable emulsions.^2^ Among the samples analyzed, crude oil F exhibited the highest interfacial tension value, 11.30 mN m^–1^. However, it is important to emphasize that these measurements may be influenced by demulsifiers used during dehydration, as well as by residual additives employed in crude oil production.^34^

In terms of dynamic viscosity, the crude oils exhibited low viscosities in the range of 11–35 mPa·s at 40 °C. With respect to the highest pour point, the samples showed values between −9 and 15 °C, a range consistent with materials that display moderate crystalline structure formation upon cooling.^35–37^ The formation water separated from the crude oils exhibited slightly acidic pH values, between 6.0 and 6.8.

Compositional analysis of the crude oils included the determination of the percentages of saturates (S), aromatics (A), and polars (P), the latter corresponding to resins and asphaltenes. The results indicated that oil D was the most apolar sample, i.e., it showed the highest API gravity and the greatest proportion of saturated (paraffinic) compounds. In contrast, crude oils B, C, and F can be grouped in decreasing order B > C > F with respect to their polar compound content and TAN values.

### GC-FID, CHNS-O, and FTIR

The comparison between the GC-FID chromatograms of the IMW (Figure 2a) and IMR (Figure 2b) fractions reveals marked differences in the compositional profiles of the IM. The fractions obtained by the wet silica method (IMW) did not exhibit the characteristic distribution of *n*-paraffins, confirming the selective nature of this method for retaining polar compounds. In contrast, the fractions isolated by the centrifugation/Dean–Stark procedure (IMR) displayed well-defined *n*-paraffin distributions spanning the C_10_–C_30_ range. These results suggest that the wet silica method tends to underestimate the contribution of apolar species such as waxes, which have been identified as potential contributors to rigid interfacial structures.^38,39^ On the other hand, GC-FID chromatograms of the crude oils reported in the literature have shown a bimodal *n*-paraffin distribution between C_7_–C_17_ and C_18_–C_30_, together with the presence of cyclic and branched hydrocarbons (HCs).^10^ Thus, when these results are compared with the IMR fractions, a selective migration of higher–carbon-number linear HCs is observed. In particular, the IMR from crude oil B exhibits higher relative abundances of long-chain *n-*paraffins than those of the other crude oils, consistent with a waxier interfacial composition for crude oil B.

**Figure 2:**
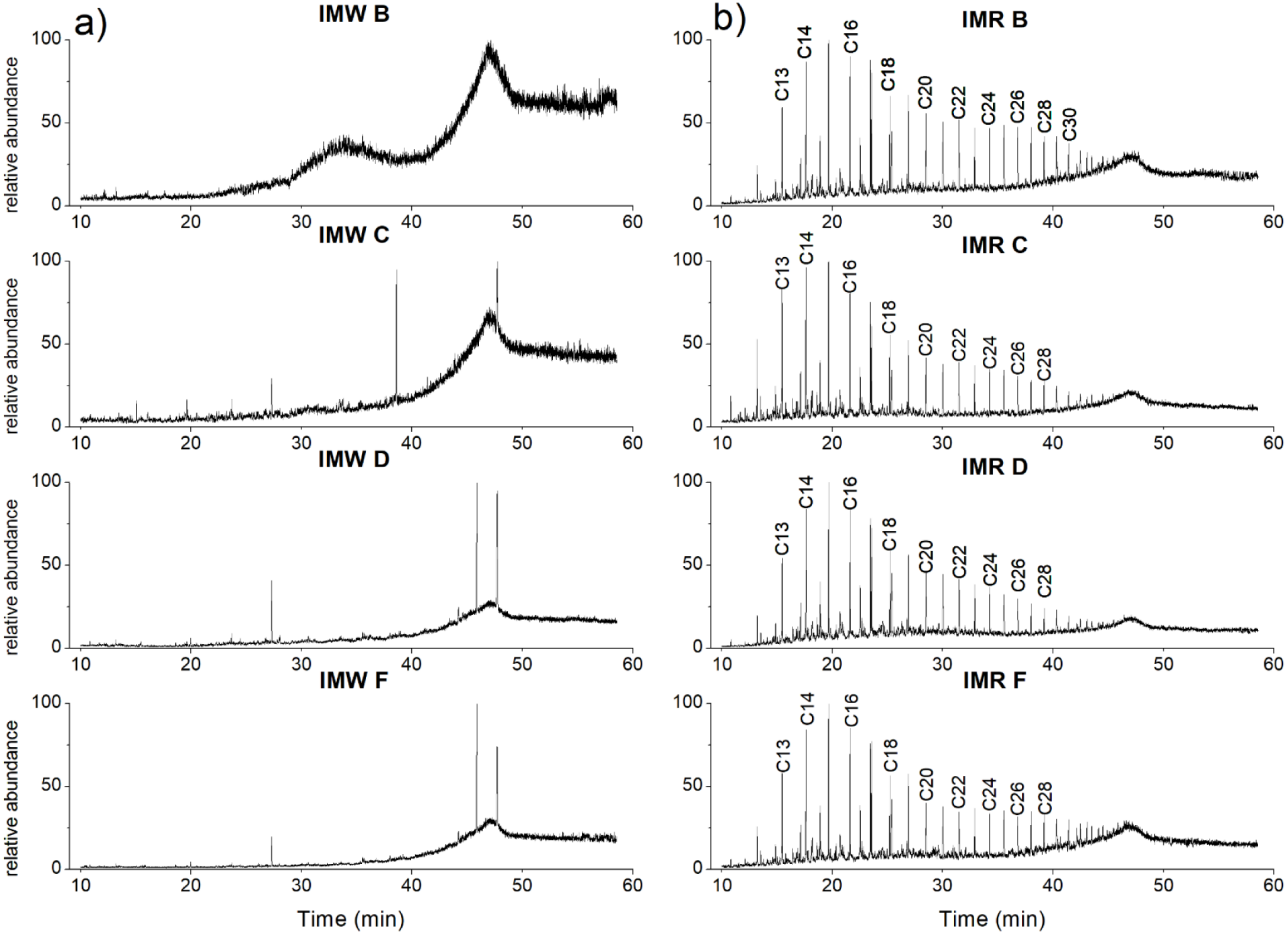
GC-FID chromatograms of interfacial material fractions obtained by (a) the wet silica method (IMW) and (b) the centrifugation/Dean–Stark method (IMR) for crude oils B, C, D, and F.

The CHNS-O results (Table S3) and the distribution shown in Figure 3 clearly distinguish the crude oils from their corresponding interfacial fractions and, in particular, highlight the selectivity of the two extraction methods. In the 3D plot, the crude oils cluster in the region of lower N/C and O/C ratios and higher H/C ratios, consistent with a poorly heteroatom composition. In contrast, the IMW fractions are displaced toward higher N/C and O/C values and lower H/C values, indicating strong enrichment in heteroatom-containing HCs species. The IMR fractions occupy an intermediate compositional region between the crude oils and IMW. This distribution is consistent with the FTIR data (Figure 4), which shows that both extraction procedures concentrate polar interfacial species relative to the crude oils. The wet silica method preferentially isolates a fraction enriched in oxygen- and sulfur-containing compounds, whereas the centrifugation/Dean–Stark method recovers an interfacial fraction with a compositionally balanced chemical profile.^10,40^ Thus, Figure 3 not only separates the three groups visually, but also reinforces that IMW fractions emphasize the most polar and unsaturated portion of the interfacial material, while IMR preserves a fraction that remains intermediate between the original crude oil and the highly polar interfacial material isolated by silica.

**Figure 3:**
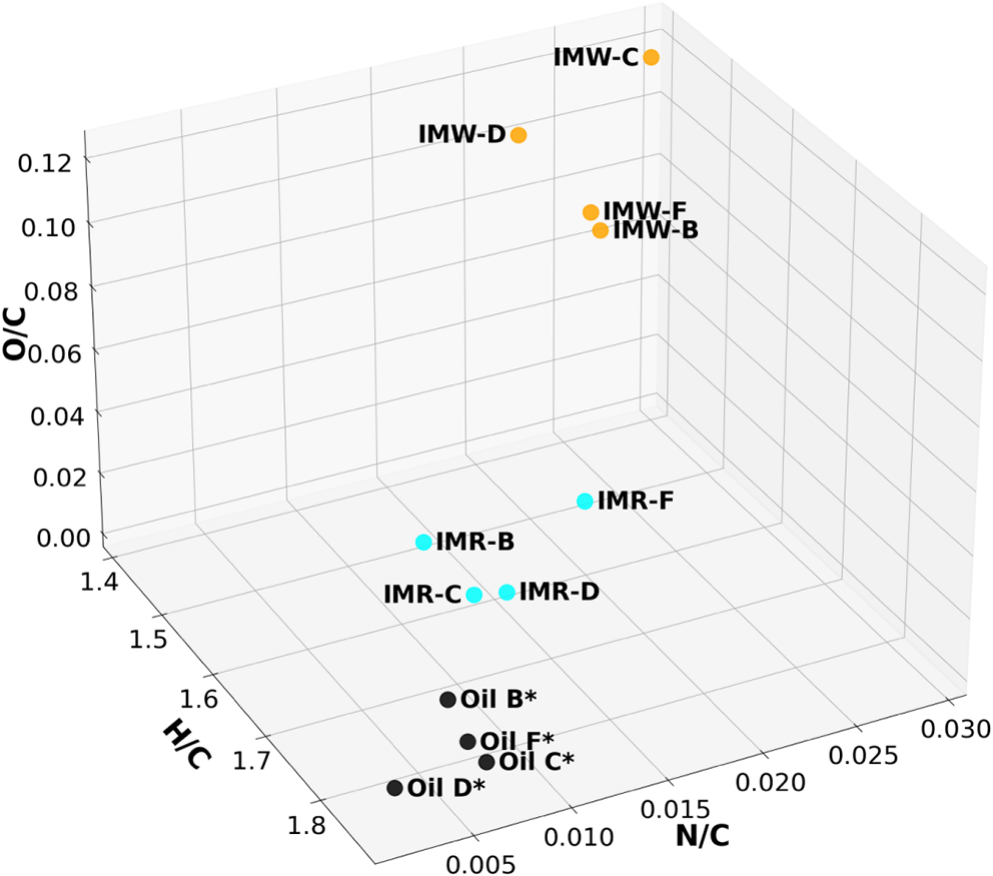
3D plot of the N/C, H/C, and O/C atomic ratios for crude oils and their corresponding interfacial materials (IMR and IMW).

**Figure 4:**
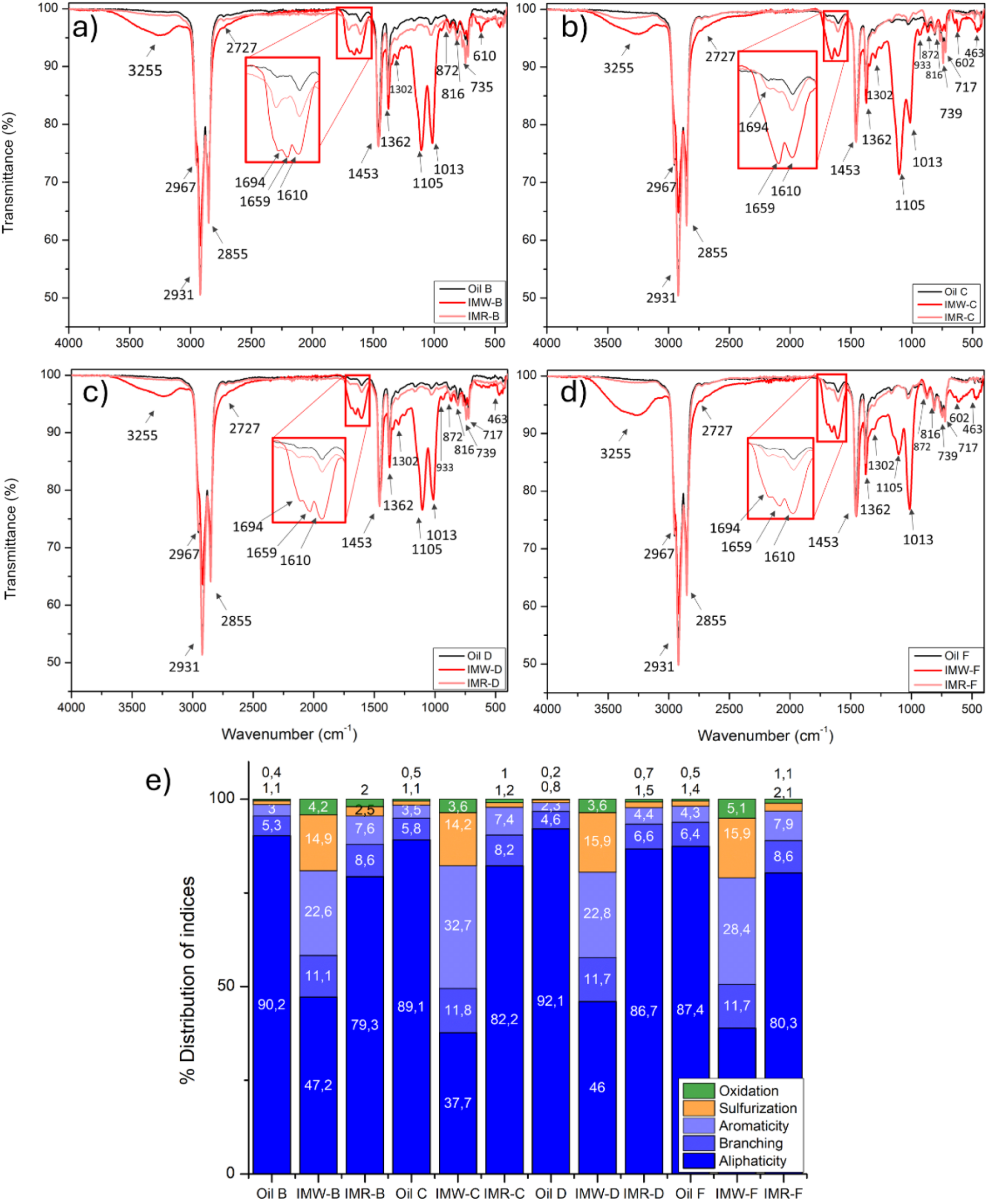
(a–d) Mid-FTIR spectra of crude oils B, C, D, and F and their interfacial materials (IMW and IMR), and (e) distribution of aliphaticity, branching, aromaticity, sulfoxide, and oxidation indices.

The mid-FTIR spectra of the crude oils (B, C, D, and F) and the corresponding IMW and IMR fractions are shown in Figure 4a–d. Overall, all samples display common bands characteristic of hydrocarbons, but with differences in the intensity and resolution of signals associated with heteroatomic compound classes. In all cases, intense bands at 2967–2855 cm^–1^ are observed, which are assigned to the asymmetric and symmetric stretching of −CH_2_ and −CH_3_ groups, consistent with the predominance of aliphatic chains in the matrix.^56–58^ However, in the interfacial fractions (IMW and IMR), additional bands in the 1694–1610 cm^–1^ region become more prominent, attributed to C=O (carbonyls, carboxylic acids) and aromatic C=C vibrations, suggesting enrichment in oxygenated and unsaturated species.^59–65^ This difference is more evident in the expanded spectra, in which IMW exhibits sharper and more intense absorptions in this region compared with the parent crude oil and IMR. Bands at 1105–1013 cm^–1^, related to S=O and C–O stretching, also increase in intensity in the IMW fractions, reinforcing the contribution of sulfur and oxygen-containing compounds at the interface,Table S2.^10,58,64,67–69^ Whereas the wet silica method (IMW) preferentially concentrates polar species, the centrifugation/Dean–Stark method (IMR) recovers a broader range of polar and apolar compounds, including those that are weakly adsorbed, which is reflected in spectra with a greater diversity of absorptions.^10,15,40,41,66^

Figure 4e shows the spectral indices derived from the mid-FTIR data. Both IMW and IMR fractions exhibit lower aliphaticity (IMW_alif = 38–47% and IMR_alif = 79–87%) and higher aromaticity (IMW_ar = 22–33% and IMR_ar = 4.4–7.9%), oxidation (IMW_oxid = 3.6–5.1% and IMR_oxid = 0.7–2.0%), and sulfoxide character (IMW_S = 14–16% and IMR_S = 1.2–2.5%) compared with the crude oils, indicating enrichment in polar and unsaturated compounds. Among these, the IMW samples display the highest oxidation and sulfoxide indices, reflecting the presence of carbonyl and sulfur-containing groups that may contribute to emulsion stability, in agreement with the elemental analysis data (Table S3 and Figure 3). In comparative terms, for the crude oils analyzed in this study, IMR recovers interfacial fractions that appear to reflect a broader portion of the interfacial composition, whereas IMW fractions are biased toward more polar compounds

The FTIR (Figure 4), GC-FID (Figure 2), and elemental analysis (Figure 3) results show convergent trends: the interfacial fractions exhibit lower aliphaticity and higher heteroatom (N, O, and S) contents, reflecting enrichment in polar and unsaturated species. The wet silica method (IMW) concentrates strongly oxygenated and sulfur-containing compounds, whereas the centrifugation/Dean–Stark method (IMR) recovers a more balanced fraction containing both saturated and polar components. These findings indicate that IMW isolates highly functionalized species, while IMR, under the experimental conditions adopted here, appears to preserve a representative portion of the chemical complexity of the oil–water interface.

## ESI(−)FT-ICR MS

To deepen the molecular identification of polar species, ESI(−)­FT-ICR MS analysis of crude oils and their respective interfacial fractions (IMW and IMR) was performed. It should be noted that the compositional profiles obtained by ESI(−)­FT-ICR MS also reflect the intrinsic ionization selectivity of the technique. In negative-ion mode, compounds bearing readily deprotonable functional groups, especially oxygenated acidic species such as carboxylic acids and phenolic compounds, tend to produce stronger signals. Figure 5 highlights the significant compositional differences resulting from the two extraction methods. The crude oils display narrow Gaussian distributions (*m*/*z* ≈ 150–500) and average molecular mass (Mw) distributions between 426 and 452 Da, consistent with the predominance of low-polarity heteroatomic hydrocarbons. In contrast, the fractions obtained by IMW and IMR, in most cases, exhibit broader Gaussian distributions (*m*/*z* ≈ 150–800) and higher Mw values (434–638 Da), indicating enrichment in polar species with higher molecular mass distribution (Mw), such as resins, asphaltenes, and compounds containing N, O, and S. For example, the number of assigned compositions (AC) increases substantially from crude oil F (AC = 1,473) to the IMR-F fraction (AC = 4,495), reaching a maximum of AC = 7,235 for the IMW-F fraction. The same trend is also observed for samples B, C, and D, confirming the method’s selectivity for enriching highly polar species. This behavior is in agreement with previous studies. Clingenpeel et al. (2017) demonstrated that the fractionation of Athabasca bitumen interfacial material by extraction with modified aminopropyl silica (MAPS) followed by ESI(−)­FT-ICR MS analysis resulted in an increase in the number of assigned formulas (∼8,000) and the mass range (∼850 Da, centered at ∼425 Da).^30^ Wu et al. (2022) showed that IM isolated from different crude oils by the wet silica method and characterized by ESI-Orbitrap MS exhibited broader mass distributions (*m*/*z* 200–800) and enrichment in multiheteroatomic species (classes O_x_, O_3_S_1_, O_4_S_1_, and N_1_O_2_), in contrast with the predominantly hydrocarbon profile of the corresponding crude oils.^14^ These results corroborate the increase in *M*_w_, the number of assigned formulas, and the enrichment in N, O, and S-containing compounds observed for the IMW and IMR fractions in this study.

**Figure 5:**
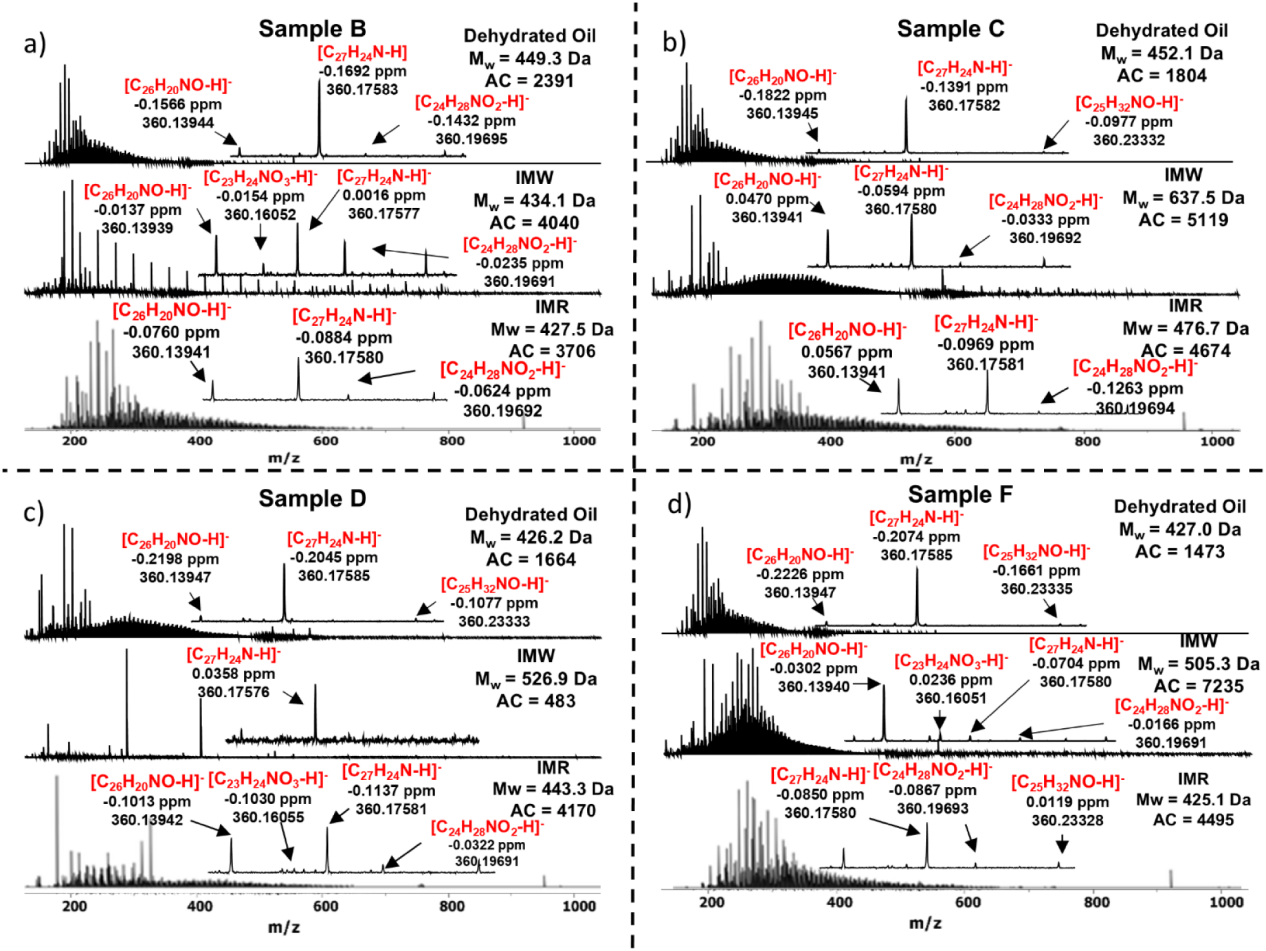
ESI­(−)­FT-ICR MS spectra of crude oils B, C, D, and F and their respective fractions obtained by wet-silica extraction and centrifugation/Dean–Stark. The zoomed-in regions show the experimental mass values, mass error (in ppm), and the molecular formula of species from the N­[H], NO_x_[H], and NO_x_S­[H] classes identified within the *m*/*z* range of 360.0 to 360.2.

In general, it is not possible to determine which method for obtaining the interfacial material yields a greater quantity of polar species, since this characteristic intrinsically depends on the crude oil under investigation. However, the IM samples obtained from crude oils B and D by both methods showed relatively similar numbers of assigned species, with AC values ranging from 4,040 to 5,119. In the case of crude oil F, the wet-silica system resulted in a significantly higher number of identified species (AC = 7,235) compared to the centrifugation/Dean–Stark method (AC = 4,495). This finding indicates that the efficiency of the extraction method may vary according to the chemical specificity of the crude oil.

Figure 6 presents the relative abundance histograms of the molecular classes identified by ESI(−)­FT-ICR MS for the crude oils and their respective interfacial fractions obtained by the wet-silica (IMW) and centrifugation/Dean–Stark (IMR) methods. In all samples, the N­[H] class, associated with pyrrole-like compounds, shows high abundance in both the crude oils and their corresponding interfacial fractions, reflecting the presence of basic nitrogen-containing species of the pyrrolic type, which are typical of petroleum.^4^ Overall, both methods produced similar class distribution profiles. However, the IMW method appears to be slightly more selective for extracting highly oxygenated compounds (O­[H], O_2_[H], O_3_[H], NO­[H], and NO_2_[H]), whereas the IMR method resulted in IMs with a broader range of mixed classes, including N_2_[H], N_2_O­[H], N_2_O_2_[H], NO­[H], NO_2_[H], NO_3_[H], O­[H], O_2_[H], O_3_[H], and O_3_S­[H].

**Figure 6:**
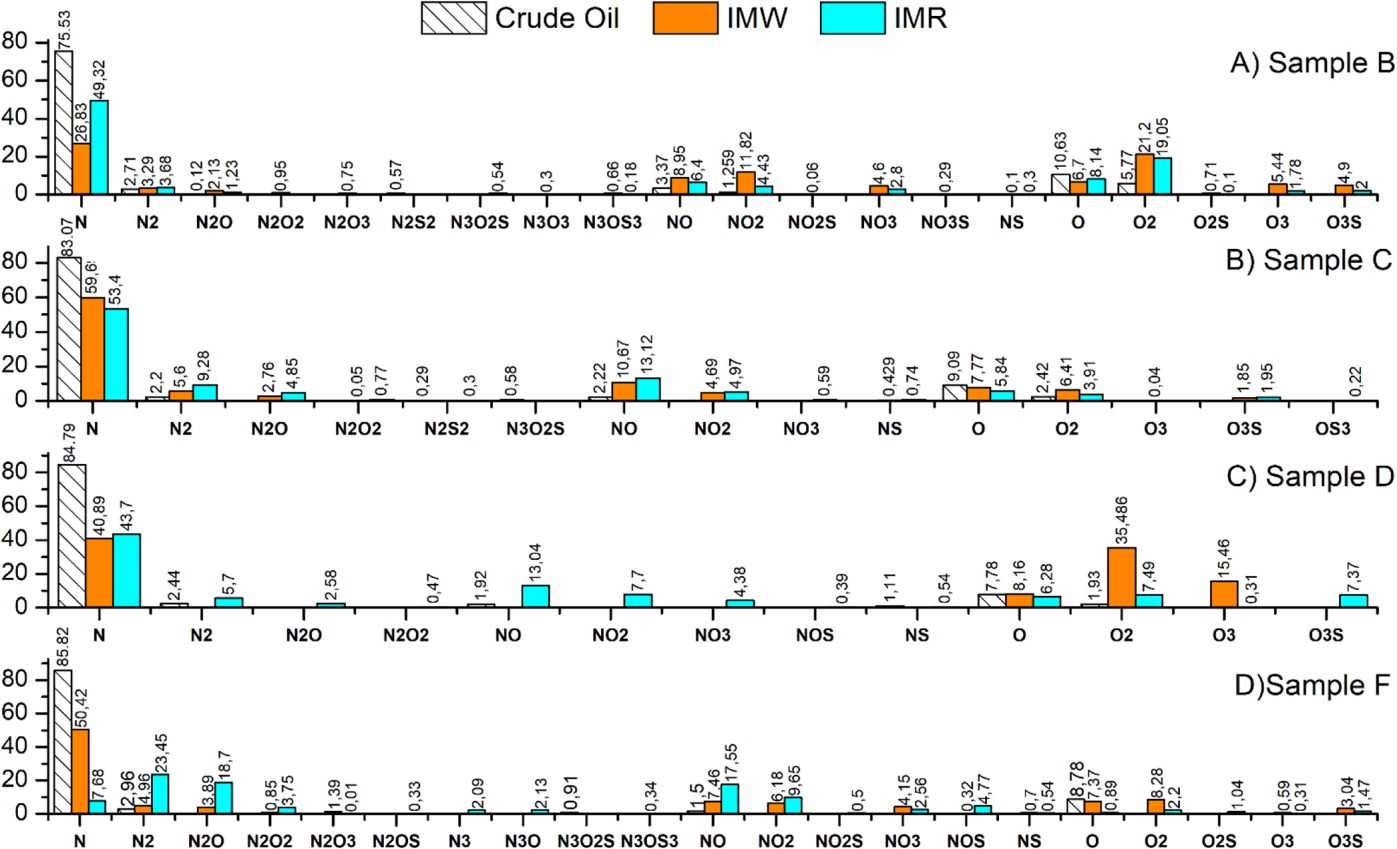
Class distribution plot of heteroatom-containing compound classes for crude oils (a) B, (b) C, (c) D, and (d) F, and their respective IMW and IMR fractions.

Among the species identified in the IMW and IMR fractions, the enrichment in the O_2_[H], O_3_[H], NO­[H], N_2_O­[H], NO_2_[H], and O_3_S­[H] classes is noteworthy when compared to their original crude oils. For example, in the wet-silica method, this selective recovery of oxygenated compounds is associated with the ability of silica to adsorb higher-polarity molecules, reinforcing the role of these species in the formation of stable interfacial films.^15,42^ In addition, the higher abundance of these classes in ESI(−) should also be interpreted in light of the greater ionization efficiency of deprotonable oxygen-containing functionalities, particularly acidic species. Therefore, the prominence of these classes reflects both extraction selectivity and the intrinsic response of negative-ion electrospray ionization mode. The exclusive detection of the O_3_S­[H] class in the samples (with formulas C_17_H_38_O_3_S, C_18_H_30_O_3_S, and C_19_H_32_O_3_S) indicates the retention of alkylbenzenesulfonates, possibly originating from native sulfonates or field additives.^1,30,43,44^

The DBE versus relative abundance diagrams (Figure 7) for the O­[H] class show that, in the crude oils analyzed, the DBE range is concentrated between 4 and 14. However, for the IMW and IMR fractions, the interval generally expands to DBE = 1–17. Chemically, the higher intensity of species with DBE > 10 in the IM fractions may indicate that the interface is enriched in more aromatic and/or more condensed species than the crude oil. IMW captures polar compounds strongly bound to the interface, whereas IMR reveals both strongly and weakly bound compounds. According to previous studies, oxygen-containing molecules (phenols, naphthenophenols, or alcohols) promote hydrogen bonding and van der Waals interactions (π–π stacking and solvophobic effects), favoring aggregation and the formation of gel-like networks. The alignment of paraffins with the alkyl chains of these species reinforces emulsion gels and affects rheological properties.^15,38^ Wax crystals may also adsorb secondarily at the interface, acting as Pickering-type stabilizers, in which solid particles, upon anchoring at the W/O boundary, form a rigid and energetically stable physical barrier that prevents the approach and coalescence of droplets, thereby enhancing the mechanical resistance of the interfacial film and altering the rheological behavior of emulsions. At low temperatures, fine crystals precipitate and form a three-dimensional network that increases crude oil viscosity, reduces the mobility of water droplets, and delays their coalescence, while residual crystals at the interface provide an additional physical barrier that preserves emulsion integrity.^45^

**Figure 7:**
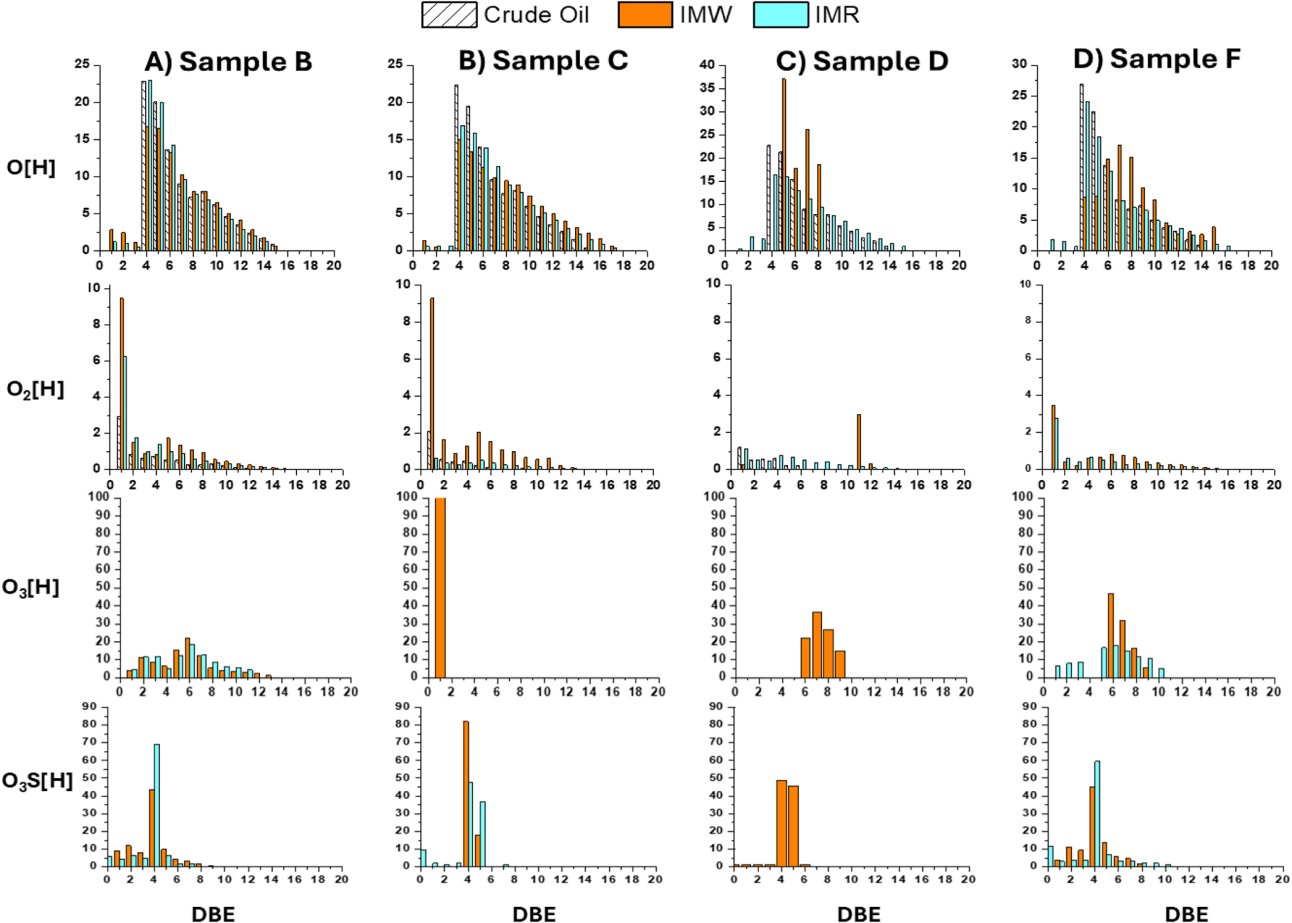
DBE versus intensity plots for the classes: (1) O­[H], (2) O_2_[H], (3) O_3_[H], and (4) O_3_S, for the crude oil samples A) B, B) C, C) D, and D) F, and their respective IMs obtained by the wet-silica and centrifugation/Dean–Stark methods.

In the O_2_[H] class histograms (Figure 7), all crude oils except crude oil F show a strong predominance at DBE= 1, typical of monocarboxylic acids (linear NAs) and, to a lesser extent, monocyclic naphthenic acids. The DBE distributions range from 1–6 for crude oils C and D, and from DBE = 1–13 for crude oil B. However, these species are not detected in crude oil F.

When moving to the IM fractions, similarities between the extraction methods emerge. The fractions retain a DBE range of 1–15, with higher abundance at DBE = 1 and DBE = 4 and 6, which may be associated with the presence of aliphatic monocarboxylic acids (DBE = 1), cyclic acids (DBE = 4), and/or aromatic acids (DBE ≥ 5) in the interfacial film. Thus, this profile is consistent with the behavior already observed for the O_2_[H] class, whose migration dynamics depend on both polarity and structural complexity. Initially, low-Mw acids with DBE = 1 predominate; their high mobility and more hydrophilic character facilitate rapid adsorption at the interface, reducing interfacial tension.^16,18^ As the process progresses, more unsaturated species, including monocyclic and polycyclic compounds (DBE ≥ 4), begin to accumulate, reflecting their lower mobility and increasing affinity for the interfacial phase. This progressive enrichment of the O_2_[H] class reinforces the hierarchical nature of the process: first, the interface becomes saturated with lower-mass linear acids, followed by the gradual incorporation of more aromatic and higher-DBE structures, which contribute to increased rigidity and cohesion of the interfacial film.^16^

The class assigned to alkylbenzenesulfonates (O_3_S­[H] class, Figure 7) was detected only in the IM fractions, with DBE distributions ranging from 1 to 10 in samples B and F and from 1 to 6 in samples C and D. Previous studies have also reported O_3_S­[H] species in interfacial materials, suggesting their affinity for the oil–water interface,^30,43,46^ These sulfur-containing compounds have been discussed in the literature as potentially acting together with naphthenic acids (O_2_[H] class), resins, and asphaltenes in the formation or maintenance of interfacial films, thereby contributing to emulsion stability.^46^ However, because compounds assigned to this class may also originate from exogenous sources, such as synthetic surfactants or contamination, their interpretation as intrinsic constituents of the interfacial material should be made with caution.

The DBE versus relative abundance diagrams for the N­[H] and N_2_[H] classes (Figure S4), and NO­[H], NO_2_[H], and NO_3_[H] classes (Figure S5), for the crude oils and their corresponding fractions obtained by the wet-silica and centrifugation/Dean–Stark methods, are provided in the Supporting Information. In general, the DBE ranges observed in the IMW and IMR fractions were broader than in the corresponding crude oils, indicating a preferential extraction of highly aromatic species. In the IMW set, the N­[H], N_2_[H], NO­[H], and NO_2_[H] classes exhibited more extensive DBE profiles. The N_2_[H] and NO_2_[H] classes were detected in all samples except IMW D. Finally, the NO_3_[H] class was observed only in IMW B and IMW F.

In contrast, in the IMR fractions, the NO­[H], NO_2_[H], and NO_3_[H] classes were consistently observed in all samples, indicating that the centrifugation/Dean–Stark procedure is more reproducible than the wet-silica method and enhances the recovery and detection of these N_x_O_y_[H] species, which are predominantly present in the interfacial material. Although these compounds contain heteroatoms, some may exhibit low polarity and be eluted in fractions with weaker affinity for the interface or even in nonpolar fractions, a behavior previously reported in the literature for the identification of heteroatom classes in saturated fractions.^47^

To assess the degree of aromaticity in the DBE vs NC plots of the FT-ICR MS data and correlate this parameter with the H/C ratio from the CHNS-O analysis, the concept of the planar limit was used. This concept allows one to identify whether the extraction method preferentially isolates molecules with higher or lower aromaticity, an essential aspect for understanding emulsion stability.^48,49^

In this context, the planar limit is obtained from the maximum DBE values for a given CN. The steeper the slope of the DBE vs. CN plot, the higher the π-electron density, and consequently, the structural stability associated with aromaticity. Thus, fractions with a lower slope, such as the saturated (∼0.28), display predominantly aliphatic or saturated cyclic structures, while aromatic (∼0.73) and asphaltenic (∼0.95) fractions indicate progressive electronic conjugation and a higher number of fused benzene rings.^48,49^ The O_2_[H] class (NAs) showed consistent behavior between the two procedures for obtaining the interfacial material. In Figure 8, IMW fractions B, C, and F exhibit a wider DBE vs. CN domain than their respective source oils. Particularly, IMW F and IMR F show signs of O_2_[H] not observed in crude oil F. The slope of the line in IMW D (planar limit value ≈ 1) supports the dominant presence of aromatic NAs, and as discussed by Cho et al. (2011),^48^ slope values close to 0.95 indicate the presence of molecules with highly condensed structures, characterized by a higher number of fused benzene rings. Thus, the higher slope observed for IMW D reflects a greater degree of aromatic condensation, and the inferred order of aromaticity by the limit lines follows IMW D > IMW F > IMW B > IMW C. The results are in good agreement with the CHNS-O data, which show fractions IMW D and IMW F as the most aromatic (H/C = 1.414 and 1.415, respectively), Table S3. Similarly, the fraction with the largest CN distribution range, C_10_–C_60_ for DBE acid = 1, exhibited the highest wt % of oxygenates, equivalent to 11.7 ± 1.0 (IMW-C). Likewise, the residual IMR fractions, via centrifugation/Dean–Stark, also show broader DBE ranges than their corresponding crude oils, except for crude oil F, in which O_2_[H] class was not detected.

**Figure 8:**
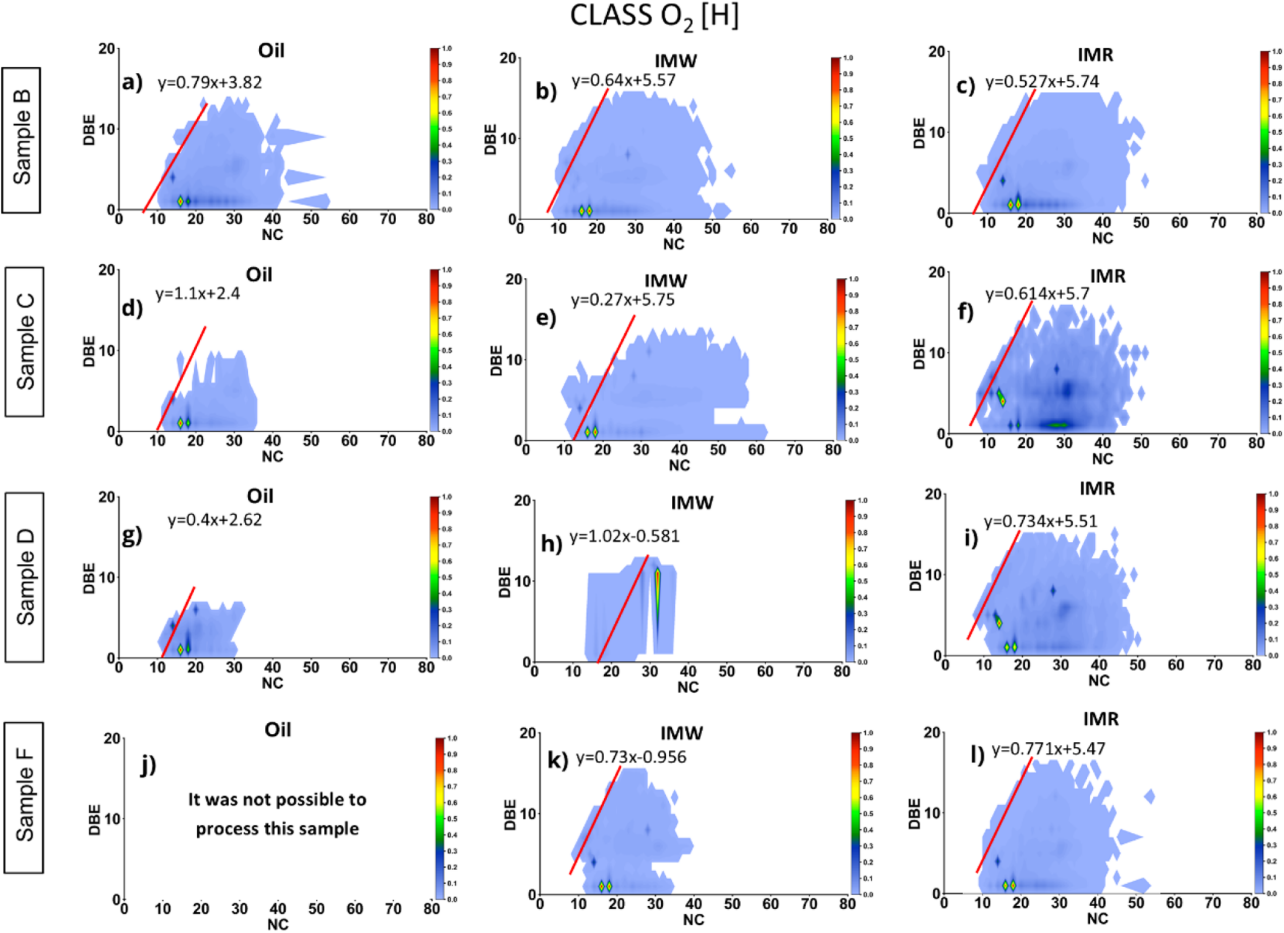
DBE versus carbon number plot for the O_2_[H] class of crude oils B, C, D, and F and their respective fractions obtained by wet silica and centrifugation/Dean–Stark methods.

The analysis of the slopes indicates that IMR D and IMR F can be classified as more aromatic, while IMR B and IMR C exhibit lower aromaticity. Considering the planar limit of the DBE × CN plot, the decreasing order of the slopes follows IMR F > IMR D > IMR C > IMR B. This trend indicates that, although fractions B and C show lower slope in this metric, the structural and association evidence mentioned above places fraction F among the most aromatic IM fractions as shown in Table S3 and Figure 6.

Understanding the role of linear acids in the formation of the interfacial film,^16^ and considering that Figure 8 highlighted the intensity of species with DBE = 1, Figure 9 presents the carbon number distribution exclusively for the linear O_2_[H]. In this distribution, a consistent pattern is observed across all samples, with a marked enrichment in C_16_–C_18_, both in the crude oil and in the IMW and IMR fractions. This behavior confirms that medium-chain NAs acids constitute a significant portion of the O_2_[H] class with DBE = 1 and, especially in the interfacial fractions, are more intense, indicating a preferential and efficient migration of them to the interface. Furthermore, long-chain NAs acids (>C_35_) are also preferably extracted, proving a greater migration of these species at the interface. This reinforce that the mobility and amphiphilic nature of these linear NAs acids may be key determinants in the interfacial stability. Together, the results highlight that linear NAs are structurally concentrated in C_10_–C_45_ chains and represent the most active and readily adsorbed portion of the O_2_[H] class in the investigated fractions.

**Figure 9:**
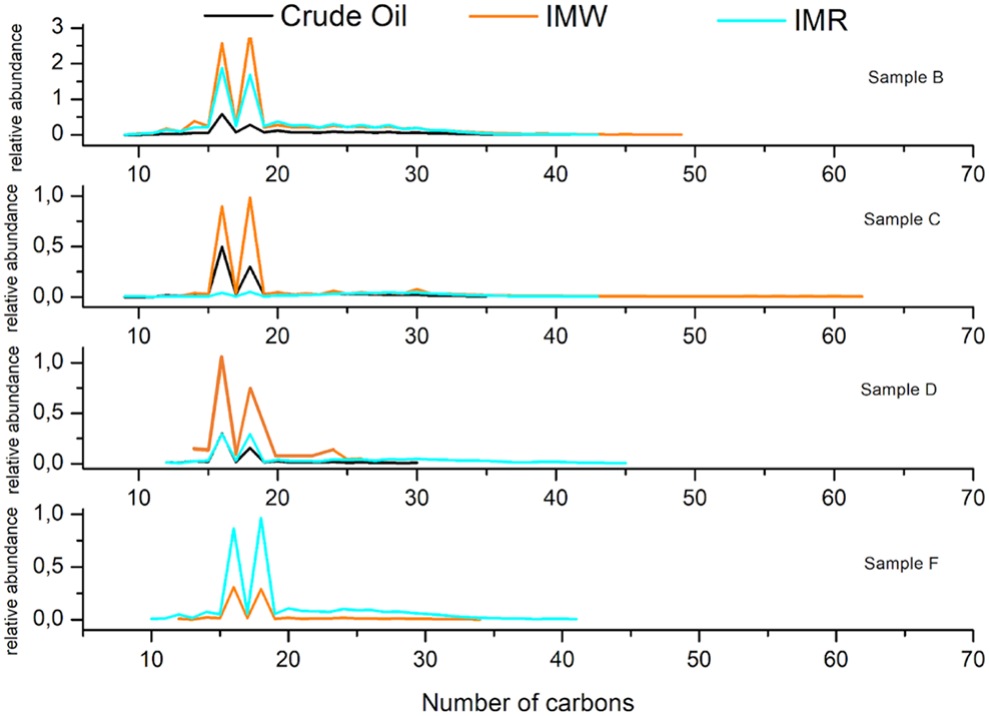
DBE 1 vs carbon number plot for the O_2_[H] class of crude oils B, C, D, F and their respective fractions obtained by silica wet extraction and centrifugation/Dean–Stark.

The Van Krevelen diagrams of the species N_x_O_y_[H] (Figure S6), obtained by ESI(−)­FT-ICR MS for IMW and IMR, show complementary results that must be interpreted in an integrated manner. In the IMW fractions, a higher abundance of the classes NO­[H] and N_2_O­[H] is observed in C and F, while for B, a reduction of all these species was verified, which are absent in the IMW D fraction. This behavior suggests a more enriched interface for fractions C and F and a less interfacially active fraction for IMW D.^48^ On the other hand, the IMR fractions suggest a greater population and homogeneity in the distribution of the species N_x_O_y_[H] classes, where NO­[H] and N_2_O­[H] are the most abundant, regardless of the crude oil, while NO_2_[H] and NO_3_[H] occur at lower levels. The IMR D fraction stands out, exhibiting a significant contribution. If the evaluation were based solely on polarity,^50,51^ the centrifugation/Dean–Stark method would suggest that IMR B and IMR D fractions have greater stabilizing capacity. However, an inverse behavior is observed for the wet silica method. In these same samples, IMW B shows a very limited distribution of the N_x_O_y_[H] species classes, while IMW D shows a complete absence of these species.

This duality indicates that each method captures distinct subsets of the interfacial material, with IMW concentrating species with high affinity for the interface, highly oxygenated species, while IMR is capable of isolating species with less affinity for the interface. Therefore, robust inferences about emulsion stability should not rely on a single interfacial material isolation method. Instead, an integrated interpretation between IMW and IMR is necessary to reflect the compositional and functional heterogeneity of the IM.^52^

### Study of Emulsion Stability

Based on a previous stability study, the W/O emulsions produced with crude oils B and C exhibited higher stability, whereas those obtained with crude oils D and F were more susceptible to destabilization.^10^ In that study, the emulsions remained stable for 2 weeks without phase separation, with average droplet sizes between 6 and 10 μm; crude oils B and C displayed a narrower droplet-size distribution, with lower polydispersity and no evidence of flocculation. Thermal stability tests showed that crude oils D and F underwent a significant increase in droplet size, indicating a loss of stability, especially for crude oil D, which was also the only sample to exhibit aqueous phase separation under centrifugation. Thus, the observed stability order was B ≃ C > F > D. In light of these stability data, some of the present results can be reinterpreted.

In crude oils B and C, the IMW and IMR fractions exhibit a broad DBE range for the O_2_[H] class, but with intermediate slopes relative to the planar limit, indicating a balance between linear species and more aromatic components. When these data are combined with the carbon number distribution at DBE = 1, which shows a pronounced enrichment in C_16_–C_18_ for both IMW and IMR, evidence emerges that these systems simultaneously concentrate medium-chain acids together with more unsaturated/aromatic species within the same O_2_[H] class.^15^ This combination is consistent with mechanisms proposed for stable emulsions, in which linear-chain carboxylic acids favor the initial adsorption at the interface, whereas more aromatic and polyacid species contribute to interfacial film thickening and gelation, enhancing its mechanical strength and retarding coalescence.^30,46,53–55^ The literature on waxy crudes further indicates that the coexistence of carboxylic acids and phenolic groups at the interface promotes the formation of rigid interfacial networks, in which paraffins align with the aliphatic chains of oxygenated compounds, trapping water and reinforcing the stability of wax-rich W/O emulsions.^15^

For crude oils D and F, Figure 8 shows IMR fractions that are apparently more aromatic within the O_2_[H] class, suggesting a greater contribution from polycyclic or condensed species. In crude oil F, this more aromatic character may be associated with the formation of a relatively rigid interfacial film that is, however, less efficient in the initial stage of interfacial coverage, which is consistent with its intermediate stability.^30,46,53–55^ In the case of crude oil D, the combination of a more aromatic IMR and an IMW that does not concentrate, or contains only low abundances of, classes potentially relevant for interfacial anchoring (such as N_x_O_y_) points to a functionally less robust interfacial film. Considering that IMW is selective for species strongly adsorbed at the interface, the lower contribution of these classes in the interfacial fractions is consistent with the higher tendency toward coalescence and phase separation observed for this crude oil.

Taken together, the results suggest that, in the systems evaluated, emulsion stability depends not only on the presence of oxygenated compounds at the interface, but on an intraclass balance between medium-chain linear O_2_[H] species, associated with interfacial anchoring and reduction of interfacial tension, and aromatic species, related to interfacial film thickening and increased film rigidity,^15,30,51,54,55^

Thus, although the correlation between the O_2_[H] distribution in the IM fractions, the presence or absence of O_x_S_y_ and N_x_O_y_ groups, the saturation/aromaticity information derived from H/C ratios obtained by elemental analysis, and the aromaticity and oxygenation indices from mid-FTIR spectra points to a trend consistent with the overall stability order (B ≃ C > F > D), it is important to recognize that not all crude oils exhibit pronounced differences in these parameters. In particular, some class distributions detected by ESI(−) FT-ICR MS showed only subtle variations, such as the low or null detection of N_x_O_y_ species in IMW-B and IMW-D and the partial similarity of O_2_[H] distributions between IMW and IMR for crude oils B and C, which limits the identification of clear trends. This limitation is consistent with previous observations that different isolation methods capture distinct subsets of the IM,^5,14,30^ Even so, when the ESI(−) FT-ICR MS data are interpreted together with CHNS-O and FTIR, the structural trends converge with the macroscopic behavior observed in the stability tests.

In this regard, crude oils B and C display a more favorable set of interfacial species, combining longer-chain *n*-paraffins, linear carboxylic acids (O_2_[H]) predominantly at C_16_–C_18_, and moderately unsaturated oxygen and nitrogen-containing species, which together contribute to the formation of more cohesive and mechanically robust viscoelastic interfacial films,^30,40,46^ By contrast, the composition of the IM associated with crude oil D-characterized by the lower recovery of N_x_O_y_ species in IMW and by the predominance of more aromatic but less diverse structures in IMR-suggests a less balanced molecular interface, with reduced efficiency in forming a highly resistant interfacial film, particularly under more severe conditions such as elevated temperatures or centrifugation. This behavior is consistent with the greater tendency toward destabilization observed experimentally and reinforces the need to consider potential analytical limitations and methodological selectivities when directly comparing molecular distributions among different crude oils.

The model system consisting of mineral oil (MO) and saline solution, as presented in Table 1, proved to be immediately unstable, exhibiting phase separation shortly after preparation. This condition remained virtually unchanged over the 10-day period, with approximately 100% of the aqueous phase separated. This behavior indicates that, in the absence of interfacially active compounds, the system has a strong tendency to undergo rapid phase separation.

**Table 1:** Results of the Room-Temperature Gravitational Separation Test for the Emulsions^a^

	**Gravitational separation (%)**				
Sample	0 h	1 h	24 h	144 h	240 h
MO	100.0	100.0	100.0	100.0	100.0
MO/IMW	10.0	40.0	40.0	40.0	40.0
MO/IMR	<2.0	<2.0	8.0	16.0	16.0

a MO: emulsion of mineral oil and brine; MO/IMW: emulsion of mineral oil with IMW and brine; MO/IMR: emulsion of mineral oil with IMR and brine.

Overall, the emulsions prepared with IM exhibited a lower extent of aqueous phase separation over time. For the emulsions containing IM obtained by the wet silica method (MO/IMW), although an initial separation was observed immediately after preparation, the amount of aqueous phase remained practically constant after the first hour and was stable throughout the entire evaluation period.

For the W/O emulsions containing IM obtained by the centrifugation/Dean–Stark method (MO/IMR), a stronger interaction between the oil and aqueous phases was observed, as reflected by the lower amount of separated aqueous phase over the course of the experiment. Consistently, the MO/IMR emulsion reached its maximum phase separation around the sixth day, at approximately 16% v/v. These results indicate that the IM obtained by the centrifugation/Dean–Stark method promotes a more intense interaction between the water and oil phases compared with the IM extracted by the wet silica method. This difference in behavior is clearly illustrated in Figure 10.

**Figure 10:**
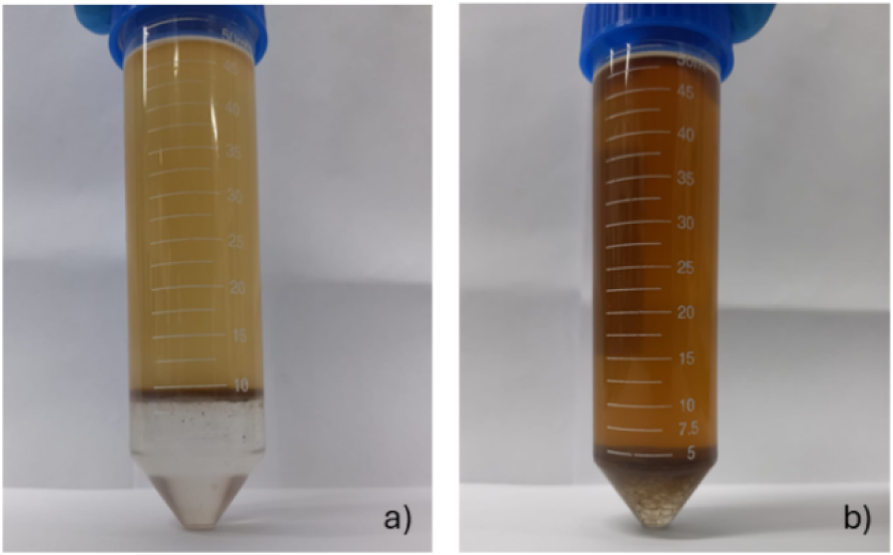
Mineral oil and crude oil emulsions containing added interfacial materials after 240 h. (a) MO/IMW emulsion; (b) MO/IMR emulsion. The mineral oil/saline system showed immediate separation (100% water). Addition of IM reduced this separation, with emulsions containing IMW stabilizing after the first hour, whereas those with IMR exhibited the lowest water release over the entire evaluation period.

The LUMiSizer dispersion analyzer enabled monitoring of phase separation kinetics through transmission profiles acquired during centrifugation, revealing clear differences in the behavior of the emulsions evaluated (Figures 11 and 12). The MO and CO/IMW emulsions, which were already unstable before the test, did not exhibit a well-defined temporal evolution. Transmittance rapidly reached high values without the formation of a continuous gradient, indicating immediate disruption of the interfacial structure and the absence of a gradual separation regime. In contrast, the mineral oil emulsion containing IMR (MO/IMR) showed a more distinct evolution, confirming that the IM obtained by the centrifugation/Dean–Stark method provides greater stability.

**Figure 11:**
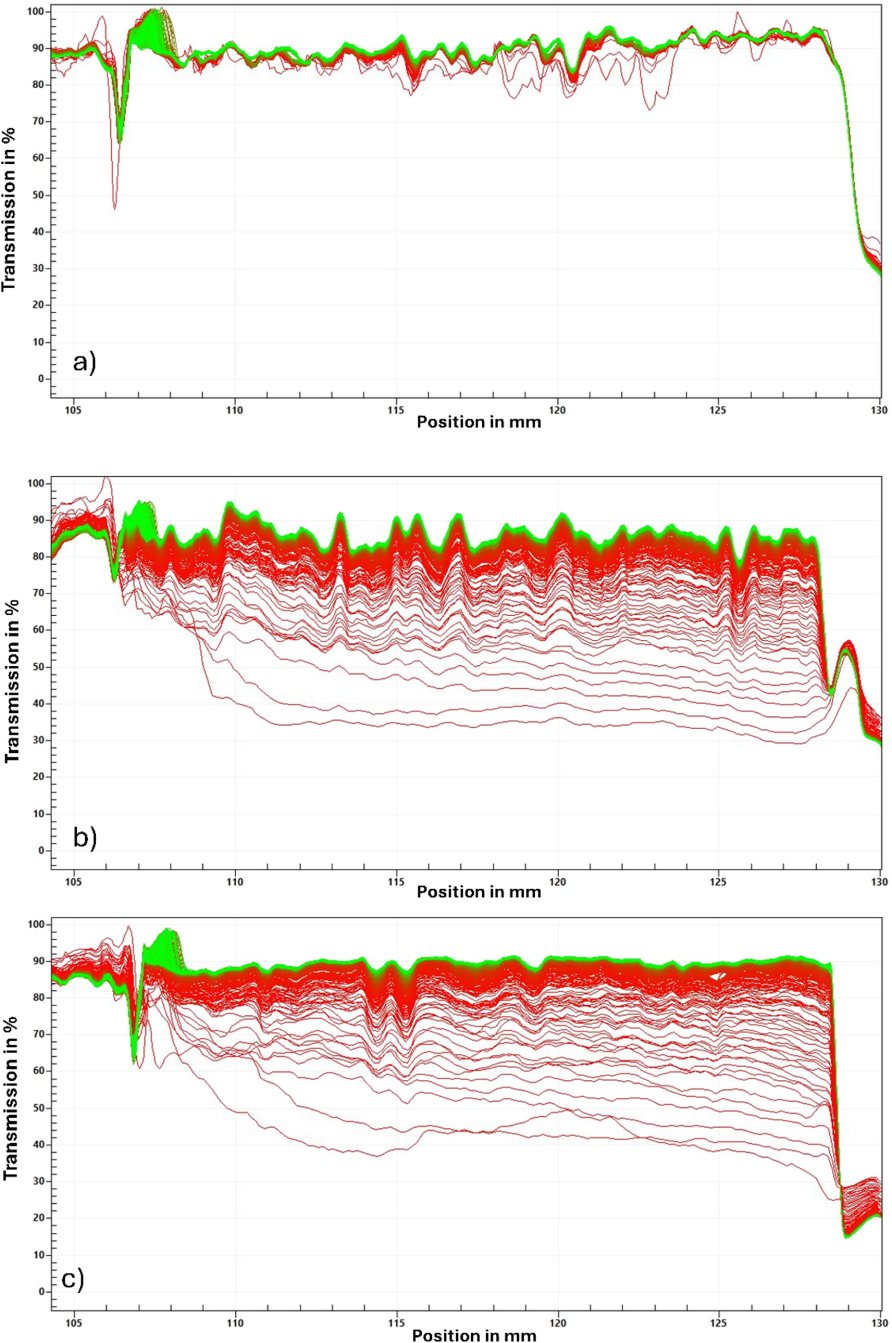
Comparison of phase separation profiles obtained with the LUMiSizer at 40 °C for W/O emulsions formulated with mineral oil and different interfacial materials: (a) MO/SS, (b) MO/IMW, and (c) MO/IMR. Each line represents one transmittance profile recorded during centrifugation; successive profiles were acquired at 5 s intervals during the first 16 min of the experiment and at 10 s intervals during the remaining time. Emulsions without IM or containing IMW showed nearly immediate breakup, whereas the system containing IMR (MO/IMR) exhibited a more gradual evolution of the transmission profiles, indicating greater stability under centrifugation.

**Figure 12:**
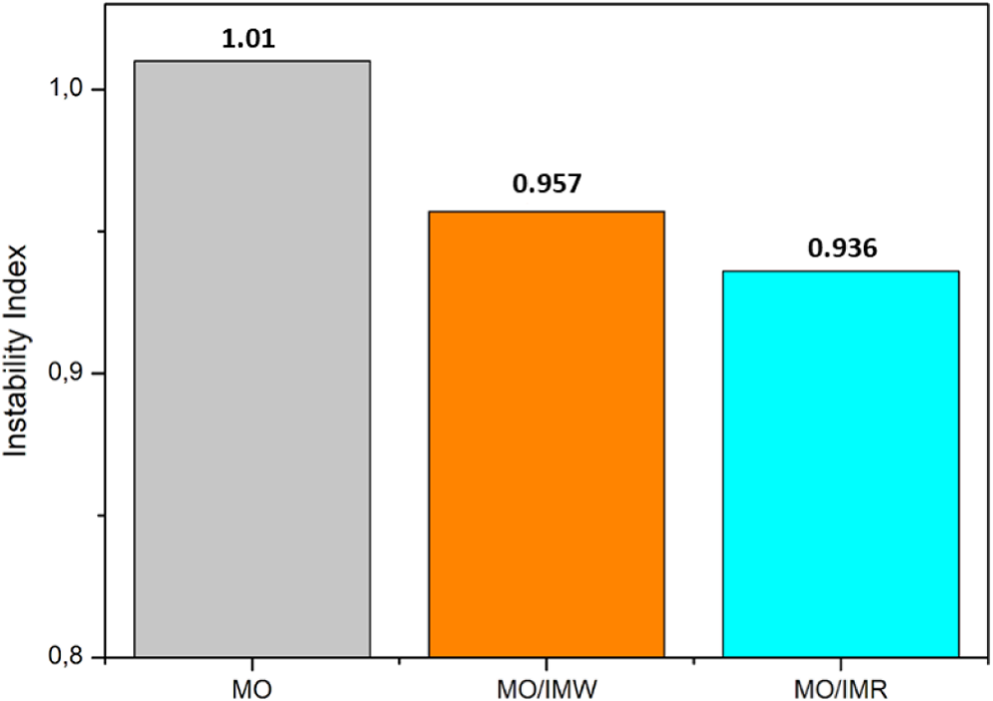
Instability index of mineral oil-based emulsions (MO) in the absence of interfacial material and in the presence of interfacial material obtained by the wet silica (MO/IMW) and centrifugation/Dean–Stark (MO/IMR) methods. The addition of IM reduced the instability index relative to the blank, with the lowest value observed for MO/IMR, indicating greater relative stability for this emulsion.

The greater stability of emulsions containing IMR can be rationalized by the nature of the interfacial material obtained by the centrifugation/Dean–Stark method. Unlike the wet silica method, which is selective for highly polar species (such as naphthenic acids, resins, and oxygenated asphaltenes), the centrifugation/Dean–Stark procedure appears to recover, for the samples analyzed here, an interfacial material fraction with broader compositional coverage, also including lower-polarity and apolar compounds, such as long-chain paraffins. Although the paraffinic species do not exhibit strong affinity for the aqueous phase, the literature indicates that they can contribute to the formation of interfacial films, imparting greater resistance to droplet coalescence and, consequently, enhancing emulsion stability,^15,38,39^ Thus, the simultaneous presence of polar natural surfactants and waxy compounds in IMR generates a more complex and stable interface, which accounts for the superior performance of these emulsions.

## Conclusion

The results obtained in this study demonstrate that the methodology employed for interfacial material isolation directly influences the observed chemical composition and, consequently, the interpretation of emulsion-stabilization mechanisms. The wet silica method proved to be selective for more polar species, enriching the IM in the O_2_[H] class, commonly associated with the presence of NAs, which are traditionally linked to the formation of rigid interfacial films. In contrast, for the crude oils investigated in this study, the centrifugation/Dean–Stark procedure preserved a more representative fraction of the interfacial material, enabling the detection of classes such as NO_2_[H] and NO_3_[H], whose lower polarity may explain their absence in the IM obtained by the silica method. The presence of the O_3_S­[H] class, also revealed by the centrifugation/Dean–Stark method, reinforces the notion that sulfur and oxygen-containing species can act in concert with resins, naphthenic acids, and asphaltenes to maintain the stability of W/O emulsions.

Moreover, GC-FID analysis showed that apolar compounds such as *n*-paraffins and waxes can also be retained in the interfacial material and indirectly contribute to the formation of the interfacial film. Stability tests confirmed that crude oil D exhibits lower resistance to coalescence, whereas crude oils B, C, and F display higher stability, likely associated with the combined contribution of polar and apolar compounds. Finally, the model emulsions (mineral oil) reinforced this interpretation, since the interfacial material obtained by centrifugation/Dean–Stark imparted greater stability than that isolated by wet silica, indicating that a full understanding of the role of IM in emulsion stability requires accounting for the simultaneous effects of polar and less polar species adsorbed at the water/oil (W/O) interface.

## Supplementary Material



## Notes

Physicochemical properties of crude oils B–D and F (Table S1); FTIR band assignments (Table S2); elemental composition and atomic ratios of crude oils and interfacial material fractions IMW and IMR (Table S3); DBE vs relative abundance plots for N­[H] and N_2_[H] classes (Figure S4); DBE vs relative abundance plots for NO­[H], NO_2_[H], and NO_3_[H] classes (Figure S5); Van Krevelen plots of N_x_O_y_[H] species for the interfacial materials of oils B–D and F (Figure S6) (PDF)
