# Supplementary material for: Chemical Insights into Interfacial Materials from Brazilian Crude Oils via Comparative Extraction Methods
Source: Langmuir. 2026 Jun 1;42(29):20911–28. doi: 10.1021/acs.langmuir.6c00674 (PMC13421996; doi:10.1021/acs.langmuir.6c00674)
Supplement: Supplementary file 1 [file la6c00674_si_001.pdf]

Supporting Information

Chemical Insights into Interfacial Materials from Brazilian Crude Oils via Comparative Extraction Methods

Thaynara R. S. Costa;[1] Bruna F. Cavalini;[2] Luciara Costa de Souza;[1] Marcos Henrique Oliveira Petroni;[1] Lays Rafalscky;[1] Amanda Eiriz Feu;[2] Boniek Gontijo;[3] Rogério Mesquita de Carvalho;[4] Osvaldo Karnitz Jr.;[4] Luiz Carlos do C. Marques;[4] Marcia Cristina Khalil de Oliveira;[4] Eliane Valéria de Barros;[2] Renato do Nascimento Siqueira;[2,5] Vitor C. B. Pegoretti;[6] Lindamara Maria de Souza[2*]; Wanderson Romão[1,2,6*]

[1] *Federal University of Espírito Santo, 29075-910 Vitória, Brazil*

[2] *Federal Institute of Education, Science and Technology of Espírito Santo, 29040-780 Vitória, Brazil*

[3] *Federal University of Goias, Goiania 74.690-631, Brazil*;

[4] *Petróleo Brasileiro S.A., CENPES, 21941-915 Rio de Janeiro, Brazil*

[5] *Federal Institute of Education, Science and Technology of Espírito Santo, 29932-540 São Mateus, Brazil*

[6] Federal Institute of Education, Science and Technology of Espírito Santo *29075-910*, Vila Velha, Brazil

Corresponding author: *wanderson.romao@ifes.edu.br (W. Romão)/ Phone: +55-27-3149-0833

Number of pages: 8

Number of figures: 3

Number of tables: 3

Summary

S1. Physicochemical properties of crude oils B, C, D and F (standard deviations in parentheses)……………………………………………………………………………………..02

S2. Assignments for the bands observed in the FTIR……………………………………04

S3. Elemental composition (wt%) and atomic ratios (N/C, H/C, and O/C) of crude oils and interfacial material (IMR and IMW)…………………………………………………………….....05

S4. DBE *vs.* relative abundance plot for the N[H] and N_2[H] classes of crude oils B, C, D, and F and their respective interfacial materials……………………………………………………...06

S5. DBE *vs.* relative abundance plot for the NO[H], NO_2[H], and NO_3[H] classes of crude oils B, C, D, and F and their respective interfacial materials………………………………………………07

S6. Van Krevelen plot N_xO_y[H] of the interfacial materials (IMW e IMR) of oils B, C, D, and F……08

S1. Physicochemical properties of crude oils B, C, D and F (standard deviations in parentheses).

Properties	Oils				Method
	B	C	D	F	
**BS&W (%v/v)**	54.4 (0.1)	8 (-0.2)	54 (-1.8)	3.64 (-0.2)	ASTM D4007 [18]
**Free Water(%v/v)**	<0.05	<0.05	20.32	<0.05	Gravitational separation [18,57]
**Sediments (%v/v)**	0.4 (0.05)	<0.05	<0.05	<0.05	ASTM D4007 [18]
**Water content in oil after dehydration (%v/v)**	0.206 (-0.0247)	0.1	0.0714 (0.0274)	0	Dehydration ASTM D4377[19,57]
**API gravity at 60 °F**	25.6 (1)	25.6 (-1)	28.2 (-1)	25.1 (-1)	ASTM D1250[20]
**Dynamic viscosity at 40 °C (mPa.s)**	34.949 (0.060)	29.30 (-0.195)	11.3605 (-0.0742)	23.659 (-0.011)	ASTM D7042 [22]
**Point of Maximum Fluidity (°C)**	9 (3)	15 (3)	9 (3)	-9 (3)	ASTM D5853 [36]
**TAN (mg de KOH·g^{-1})**	0.389 (0.001)	0.3822 (0.0241)	0.1978 (0.0019)	0.375 (0.0262)	ASTM D664 [23]
**TSI (mg•kg^{-1} de NaCl) óleo**	3500 (78) 145.000 (7426)	2885 (2.0)	16646.0 (20.0)	8658	ASTM D6470 [24]
**pH in oil dehydration water at 25 °C**	6.39 (-0.24)	6.1 (-0.12)	6.18 (-0.1)	6.18 (-0.2)	pHmetry

Properties	Oils				Method
	B	C	D	F	
**pH in oil wash water at 25 °C**	6.5 (-0.21)	6.23 (-0.1)	6.32 (-0.14)	6.21 (-0.13)	pHmetry
**Interfacial tension (mN m^{-1}) *vs* Deionized water**	6.74 (0.70)	6.42 (-0.2)	6.83 (-0.3)	11.95 (0.53)	Pendant drop [25]
**Interfacial tension (mN m^{-1}) *vs* Formation water**	4.26 (0.12)	2.54 (-0.4)	3.06 (-0.53)	11.30 (1.51)	Pendant drop [25]
**Mean droplet diameter from the Droplet Size Distribution (DSD) (µm)**	4.3 (0.4)	< 1	3.5 (-0.3)	3.6 (0.85)	Optical microscopy [25]
			**SAP**		
**Saturated (S) (wt%)**	42.29	42.6	48.23	43.1	Modified ASTM D2549 [26]]
**Aromatics (A) (wt%)**	24.98	25.45	26.06	25.12	Modified ASTM D2549 [26]
**Polars (P) (Resins +Asphaltenes) (wt%)**	32.72	31.93	20.21	31.76	Modified ASTM D2549 [26]

S2. Assignments for the bands observed in the FTIR.

Wavenumber (cm^{-1})	Vibrational modes	Assignment	References
3255	O-H<br>Axial deformation	alcohol or phenol	[70]
2854<br>2922<br>2953	C-H<br>Axial deformation	alkanes	[57-59]
2727	C-H stretch of the –CHO group	Aldehyde	[61]
1694	C=O<br>Axial deformation	ketone, ester, or carboxylic acid	[60]
1659	C=O of more conjugated groups	carboxylic acids/esters/ketones	[64]
1610	C=N<br>C=C<br>Axial deformation	Aromatics*	[60-64]
1517	C=C<br>Axial deformation	Aromatics	[42]
1453	C-H<br>Asymmetric angular deformation	alkenes	[42,59,65]
1362	C-H<br>Symmetric angular deformation	alkanes	[65,66]
1302	C–O stretching	phenolic, ether or ester-type groups	[67]
1264	C-O-C<br>Asymmetric axial deformation	aromatic ester	[62]
1158	Stretching of the –CH group	Methylene group	[42,59,65]
1135	S═O	Sulfoxide	[10,68]
1105	C–O	Primary alcohols	[42,59,65]
1013	C-O-C<br>Symmetric axial deformation	aliphatic ester	[60,69]
874<br>816	=C-H<br>Out-of-plane deformation	Aromatics with one and two adjacent H	[42]
717	C-H<br>Angular deformation (Rocking mode)	Long chain CH_2	[70]

*Benzene, substituted aromatics, and heteroatom-containing aromatic compounds, such as pyridines and pyrazines

S3. Elemental composition (wt%) and atomic ratios (N/C, H/C, and O/C) of crude oils and interfacial material (IMR and IMW).

Sample	C %	H %	N %	S %	O %	N/C	H/C	O/C
Oil B*	84.0 ± 2.8	12.3 ± 0.5	0.8 ± 0.3	-	0.5 ± 0.2	0.008	1.751	0.004
Oil C*	76.1 ± 3.4	11.8 ± 0.6	0.6 ± 0.1	-	0.6 ± 0.1	0.007	1.851	0.006
Oil D*	78.3 ± 1.1	12.2 ± 0.2	0.2 ± 0.1	-	0.9 ± 0.1	0.002	1.863	0.009
Oil F*	79.0 ± 1.8	12.1 ± 0.1	0.6 ± 0.2	-	0.7 ± 0.01	0.007	1.819	0.006
IMW-B	79.0 ± 1.0	9.9 ± 0.1	2.2 ± 0.1	3.1 ± 1.2	8.7 ± 0.5	0.024	1.495	0.083
IMW-C	73.0 ± 2.0	8.7 ± 0.03	2.4 ± 0.2	3.9 ± 0.5	11.7 ± 1.0	0.029	1.424	0.120
IMW-D	74.5 ± 2.0	8.8 ± 0.2	1.9 ± 0.1	3.7 ± 0.9	10.3 ± 0.2	0.022	1.414	0.103
IMW-F	70.8 ± 0.2	8.4 ± 0.4	2.2 ± 0.3	3.4 ± 1.7	6.8 ± 0.2	0.026	1.415	0.073
IMR-B	92.4 ± 0.9	12.2 ± 0.2	1.3 ± 0.2	-	1.7 ± 0.2	0.012	1.573	0.014
IMR-C	89.5 ± 1.7	12.2 ± 0.2	1.3 ± 0.2	-	0.7 ± 0.1	0.013	1.630	0.006
IMR-D	86.3 ± 1.2	12.0 ± 0.2	1.5 ± 0.2	-	1.1 ± 0.2	0.014	1.655	0.010
IMR-F	89.6 ± 1.6	11.8 ± 0.2	2.2 ± 0.6	-	1.4 ± 0.2	0.021	1.566	0.012

*dehydrated

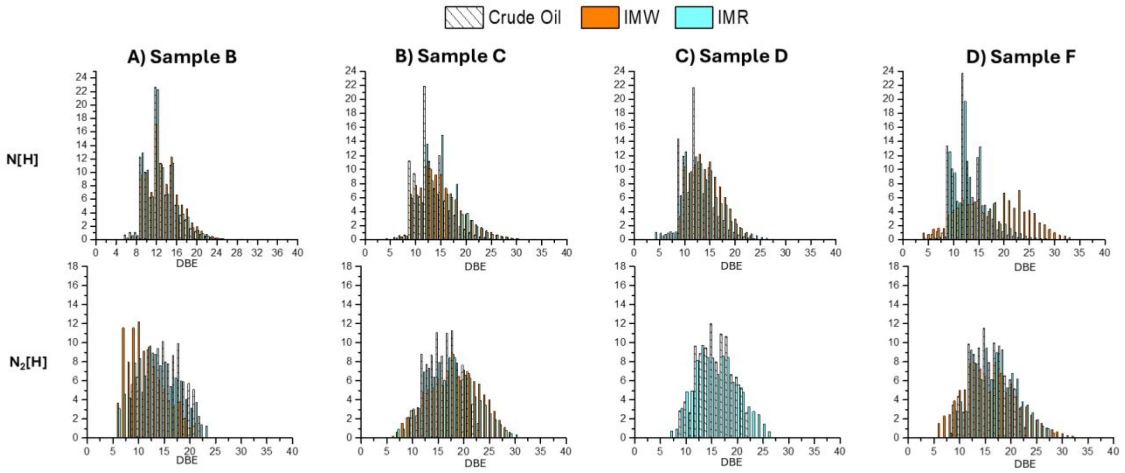

S4. DBE *vs.* relative abundance plot for the N[H] and N_2[H] classes of crude oils B, C, D, and F and their respective interfacial materials.

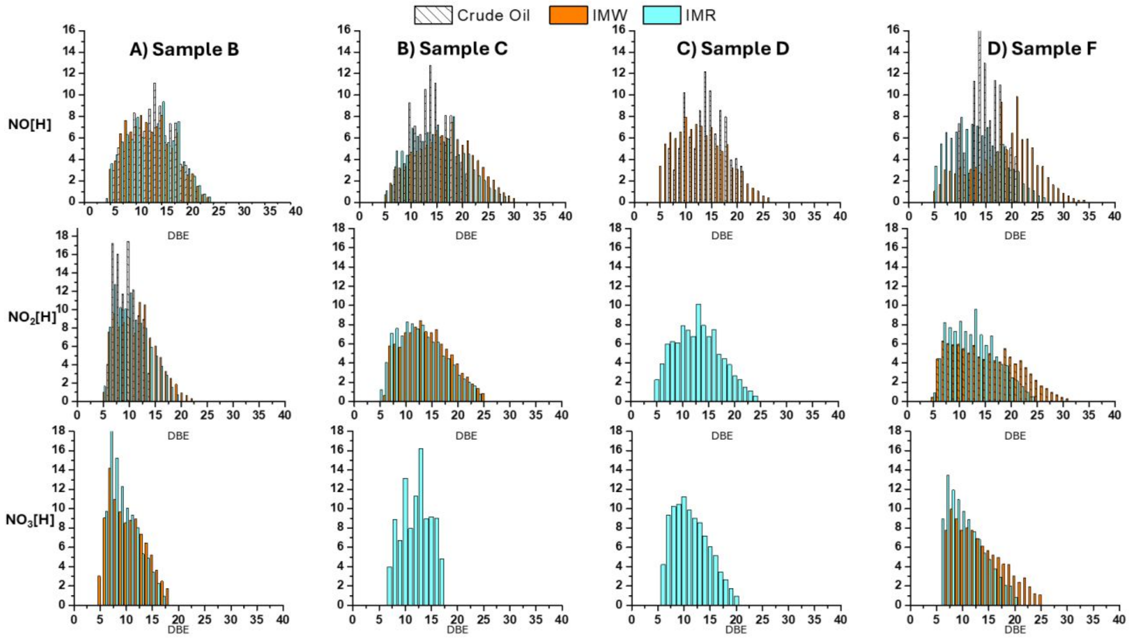

S5. DBE *vs.* relative abundance plot for the NO[H], NO_2[H], and NO_3[H] classes of crude oils B, C, D, and F and their respective interfacial materials.

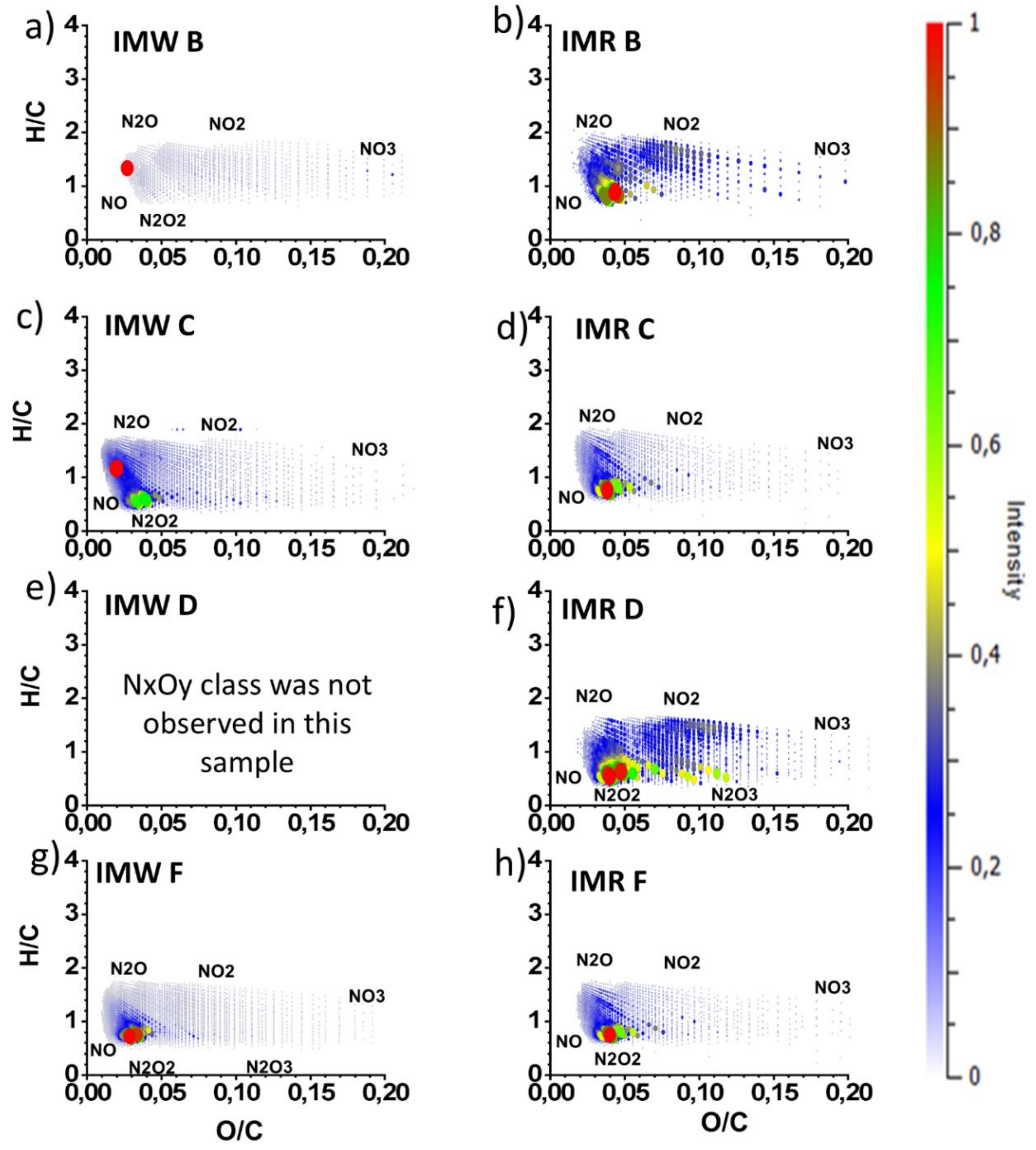

S6. Van Krevelen plot N_xO_y[H] of the interfacial materials (IMW e IMR) of oils B, C, D, and F.

REFERENCES

(18) American Society for Testing and Materials. ASTM D4007: Standard Test Method for Water and Sediment in Crude Oil by the Centrifuge Method (Laboratory Procedure). In *Manual on Hydrocarbon Analysis, 6th Edition*; ASTM International: West Consho hocken, PA, 2011; pp 606-606–611. https://doi.org/10.1520/MNL10924M.

(19) American Society for Testing and Materials. ASTM D4377. Standard Test Method for Water in Crude Oils by Potentiometric Karl Fischer Titration. In *Manual on Hydrocarbon Analysis, 6th Edition*; ASTM International: 100 Barr Harbor Drive, PO Box C700, West Consho hocken, PA 19428-2959, 2017; pp 684-684–4. https://doi.org/10.1520/MNL10934M.

(21) American Society for Testing and Materials. *ASTM D1250: Standard Guide for Use of the Petroleum Measurement Tables.* https://store.astm.org/d1250-19e01.html (accessed 2025-04-06).

(22) American Society for Testing and Materials. *ASTM D7042: Standard Test Method for Dynamic Viscosity and Density of Liquids by Stabinger Viscometer (and the Calculation of Kinematic Viscosity)*. https://store.astm.org/d7042-21a.html (accessed 2025-04-06).

(23) American Society for Testing and Materials. *ASTM D6470: Standard Test Method for Salt in Crude Oils (Potentiometric Method)*. https://store.astm.org/d6470-99r20.html (accessed 2025-04-06).

(24) American Society for Testing and Materials. *ASTM D6470: Standard Test Method for Salt in Crude Oils (Potentiometric Method)*. https://store.astm.org/d6470-99r20.html (accessed 2025-04-06).

(25) da Silva, M.; Sad, C. M. S.; Corona, R. R. B.; Pereira, L. B.; Medeiros, E. F.; Filgueiras, P. R.; Lacerda, V.; Castro, E. V. R. Analysis of the Influence of Carbon Dioxide and Nitrogen Gases on the Stability of Heavy Oil–Water Emulsions. *Fuel* **2024**, *369*, 131696. https://doi.org/10.1016/j.fuel.2024.131696.

(26) American Society for Testing and Materials. ASTM D2549. Standard Test Method for Separation of Representative Aromatics and Nonaromatics Fractions of High-Boiling Oils by Elution Chromatography; ASTM International: West

Consho hocken, PA, USA, 2017; pp 379-379–6. https://doi.org/10.1520/MNL10892M.

(36) American Society for Testing and Materials. ASTM D5853. Practice for Mixing and Handling of Liquid Samples of Petroleum and Petroleum Products, 2019. http://www.astm.org/cgi-bin/resolver.cgi?D5854-19 (accessed 2025-04-06).

(42) Silverstein, R. M.; Webster, F. X.; Kiemle, D. J. *Identificação Espectrométrica de Compostos Orgânicos (7a*; Grupo Gen-LTC, 2010.

(57) Lopes, W. A.; Fascio, M. Esquema para interpretação de espectros de substâncias orgânicas na região do infravermelho. *Quím. Nova* **2004**, *27*, 670–673. https://doi.org/10.1590/S0100-40422004000400025.

(58) Guzmán-Osorio, F. J.; Domínguez-Rodríguez, V. I.; Adams, R. H.; Lobato-García, C. E.; Guerrero-Peña, A.; Barajas-Hernández, J. R. Classification of Petroleum Origin and Integrity by FTIR. *Egypt. J. Pet.* **2021**, *30* (2), 63–67.

(59) Madeira, N. C. L.; Ferreira, P. da S.; Allochio Filho, J. F.; Chinelatto, L. S. Jr.; Cravo, M. C. C.; Martins, A. T.; Lacerda, V. Jr.; Romão, W. Study of Thermal Aging of Model Compounds Present in Asphalt Cement by GC/MS, ESI-MS, NMR, and FTIR. *Energy Fuels* **2021**, *35* (18), 14553–14568. https://doi.org/10.1021/acs.energyfuels.1c01815.

(60) Chibiryaev, A. M.; Kozhevnikov, I. V.; Shalygin, A. S.; Martyanov, O. N. Transformation of Petroleum Asphaltenes in Supercritical Alcohols Studied via FTIR and NMR Techniques. *Energy Fuels* **2018**, *32* (2), 2117–2127. https://doi.org/10.1021/acs.energyfuels.7b01630.

(61) George, K. M.; Ruthenburg, T. C.; Smith, J.; Yu, L.; Zhang, Q.; Anastasio, C.; Dillner, A. M. FT-IR Quantification of the Carbonyl Functional Group in Aqueous-Phase Secondary Organic Aerosol from Phenols. *Atmos. Environ.* **2015**, *100*, 230–237. https://doi.org/10.1016/j.atmosenv.2014.11.011.

(62) Carvalho, V. V.; Vasconcelos, G. A.; Tose, L. V.; Santos, H.; Cardoso, F. M. R.; Fleming, F.; Romão, W.; Vaz, B. G. Revealing the Chemical Characterization of Asphaltenes Fractions Produced by N-Methylpyrrolidone

Using FTIR, Molecular Fluorescence, 1H NMR, and ESI(±)FT-ICR MS. *Fuel* **2017**, *210*, 514–526. https://doi.org/10.1016/j.fuel.2017.08.098.

(63) Sun, M.; Ma, X.-X.; Yao, Q.-X.; Wang, R.-C.; Ma, Y.-X.; Feng, G.; Shang, J.-X.; Xu, L.; Yang, Y.-H. GC-MS and TG-FTIR Study of Petroleum Ether Extract and Residue from Low Temperature Coal Tar. *Energy Fuels* **2011**, *25* (3), 1140–1145. https://doi.org/10.1021/ef101610z.

(64) Ezzat, A. O.; Al-Lohedan, H. A.; Albarqan, M. Y.; Toraba, M. A.; Faqihi, N. A. The Synthesis and Application of Novel, Star-Shaped Surfactants for the Destabilization of Water in Arabian Heavy Crude Oil Emulsions. *Processes* **2023**, *11* (11), 3162. https://doi.org/10.3390/pr11113162.

(65) Moro, M. K.; Neto, Á. C.; Lacerda, V.; Romão, W.; Chinelatto, L. S.; Castro, E. V. R.; Filgueiras, P. R. FTIR, 1H and 13C NMR Data Fusion to Predict Crude Oils Properties. *Fuel* **2020**, *263*, 116721. https://doi.org/10.1016/j.fuel.2019.116721.

(66) Feitosa, F. X.; Alves, R. S.; de Sant'Ana, H. B. Synthesis and Application of Additives Based on Cardanol as Demulsifier for Water-in-Oil Emulsions. *Fuel* **2019**, *245*, 21–28. https://doi.org/10.1016/j.fuel.2019.02.081.

(67) Hebbar, A.; Debraj, D.; Acharya, S.; Puttapati, S. K.; Vatti, A. K.; Dey, P. Deep Eutectic Solvents Interaction with Asphaltenes: A Combined Experimental and Molecular Dynamics Study. *J. Mol. Liq.* **2023**, *387*, 122627. https://doi.org/10.1016/j.molliq.2023.122627.

(68) Nyquist, R. A. *Interpreting Infrared, Raman, and Nuclear Magnetic Resonance Spectra*; Academic Press, 2001.

(69) Ezemagu, I. G.; Ejimofor, M. I.; Menkiti, M. C. Instrumental and Thermal Characterization of the Sludge Generated after Bio-Coagulation Treatment (GSABT) of Petroleum Produced Water (PW). *Results Eng.* **2021**, *9*, 100187. https://doi.org/10.1016/j.rineng.2020.100187.

(70) Filgueiras, P. R.; Sad, C. M. S.; Loureiro, A. R.; Santos, M. F. P.; Castro, E. V. R.; Dias, J. C. M.; Poppi, R. J. Determination of API Gravity, Kinematic Viscosity and Water Content in Petroleum by ATR-FTIR Spectroscopy and

Multivariate Calibration. *Fuel* **2014**, *116*, 123–130. https://doi.org/10.1016/j.fuel.2013.07.122.
